# Genome-Wide Analysis of the MADS-Box Gene Family in Holoparasitic Plants (*Balanophora subcupularis* and *Balanophora fungosa* var. *globosa*)

**DOI:** 10.3389/fpls.2022.846697

**Published:** 2022-05-31

**Authors:** Kunyu Duan, Hui Fu, Dongming Fang, Kaimeng Wang, Wen Zhang, Huan Liu, Sunil Kumar Sahu, Xiaoli Chen

**Affiliations:** ^1^Beijing Genomics Institute College and Henan Institute of Medical and Pharmaceutical Sciences, Zhengzhou University, Zhengzhou, China; ^2^State Key Laboratory of Agricultural Genomics, Beijing Genomics Institute, Shenzhen, China; ^3^China National GeneBank, Beijing Genomics Institute, Shenzhen, China

**Keywords:** parasitic plants, MADS-box gene family, gene loss, gene redundancy, flowering, holoparasitic plant, *Balanophora*, phylogeny

## Abstract

MADS-box is an important transcription factor family that is involved in the regulation of various stages of plant growth and development, especially flowering regulation and flower development. Being a holoparasitic plant, the body structure of Balanophoraceae has changed dramatically over time, and its vegetative and reproductive organs have been extensively modified, with rudimentary flower organs. Meanwhile, extraordinary gene losses have been identified in holoparasitic plants compared with autotrophs. Our study reveals that the MADS-box gene family contracted sharply in *Balanophora subcupularis* and *Balanophora fungosa* var. *globosa*, and some subfamilies were lost, exhibiting reduced redundancy in both. The genes that functioned in the transition from the vegetative to floral production stages suffered a significant loss, but the ABCE model genes remained intact. We further investigated genes related to flowering regulation in *B. subcupularis* and *B. fungosa* var. *globosa*, vernalization and autonomous ways of regulating flowering time remained comparatively integrated, while genes in photoperiod and circadian clock pathways were almost lost. Convergent gene loss in flowering regulation occurred in *Balanophora* and another holoparasitic plant *Sapria himalayana* (Rafflesiaceae). The genome-wide analysis of the MADS-box gene family in *Balanophora* species provides valuable information for understanding the classification, gene loss pattern, and flowering regulation mechanism of MADS-box gene family in parasitic plants.

## Introduction

The MADS-box gene family is involved in all stages of plant development and is one of the most thoroughly investigated gene families in plants (Theißen et al., 2016; Schilling et al., 2018). Previous research elucidated that MADS-box family plays a vital role in many developmental processes, especially in the flower organ identity, control of flowering time, vegetative development, seed and fruit development, pollen, and embryo sac formation (Smaczniak et al., 2012; Theißen et al., 2016).

MADS-box genes contain a highly conserved MADS (M) domain at the N-terminus which has a length of 50–60 amino acids, binding to CArG boxes (CC-“Adenine rich”-*GG*) (Riechmann et al., 1996; Gramzow et al., 2010). Phylogenetically, MADS-box gene family is divided into two categories: type I (SRF-like) and type II (MEF2-like), based on their conserved domain (Alvarez-Buylla et al., 2000; Parenicová et al., 2003). In plants, the length of type I genes are generally shorter and have simple structures, having only the MADS domain, which can be further classified into three clades, Mα, Mβ, and Mγ. Compared to type I genes, the type II genes tend to be more complicated consisting of MADS (M) domain, Intervening (I) domain, Keratin (K) domain, and highly variable C-terminal (C) domain, also named as MIKC-type genes (Theißen et al., 1996; Parenicová et al., 2003; Kaufmann et al., 2005; Smaczniak et al., 2012). According to the different intervening regions, the MIKC-type can be divided into two subgroups, MIKC^C^ and MIKC* (Henschel et al., 2002; Liu et al., 2018). Some reports about *Arabidopsis thaliana* showed that MIKC^C^ type genes can be further subclassified into 12 groups based on their phylogenetic relationships (Parenicová et al., 2003). During the long-term evolution of this family of genes, varying degrees of duplication events have occurred followed by subfunctionalization, which resulted in the functional diversification of MADS-box genes (Theissen and Saedler, 2001).

Flowering is a critical process that requires the cooperation and interaction of many genes in order to detect developmental and environmental cues and make decisions. Numerous studies have manifested that MADS-box genes play crucial roles in the flowering process, not only participate in regulating floral transition, floral meristem specialization, floral organ formation, and pollen growth, but are also related to root, ovule, and seed development (Michaels and Amasino, 1999; Liljegren et al., 2000; Adamczyk and Fernandez, 2009; Moreno-Risueno et al., 2010; Garay-Arroyo et al., 2013; Xu et al., 2016). Extensive research on mutants with floral organ identity defects has led to the birth of the “ABCE model,” which explains how the class A, B, C, D, and E genes co-determine floral organ identities (Riechmann and Meyerowitz, 1997; Theissen and Saedler, 2001). In *Arabidopsis*, class A [*APETALA1* (*AP1*)] and class E [*SEPALLATA1/2/3/4* (*SEP1/2/3/4*)] protein complex are involved in sepal development. Class A, class B, and class E protein complex participate in petal development. Class B [*APETALA3* (*AP3*) and *PISTILLATA* (*PI*)], class C [*AGAMOUS* (*AG*)] and class E protein complex are involved in stamen development, while class C and class E protein complex take part in carpel development. Ovule development is modulated by class D genes [*SEEDSTICK* (*STK*), *SHATTERPROOF1* (*SHP1*), and *SHP2*] (Weigel and Meyerowitz, 1994; Ehlers et al., 2016). Class E proteins are involved in the formation of tetramer protein complexes and form complexes with type A, B, C proteins (Melzer and Theissen, 2009; Pan et al., 2014). In this model, most genes belong to the type-II MADS-box family in *Arabidopsis* (Kaufmann et al., 2005). In addition, there are some other type II genes of MADS-box that are also involved in the regulation of flowering time and flower initiation, including *FLOWERING LOCUS C* (*FLC*), *SHORT VEGETATIVE PHASE* (*SVP*), *SUPPRESSOR OF CONSTANS1* (*SOC1*), *AGAMOUS-LIKE* 15 (*AGL15*), *AGL18*, *AGL24*, *MADS AFFECTING FLOWERING* (*MAF1/FLM*), etc. (Michaels and Amasino, 1999; Hartmann et al., 2000; Samach et al., 2000), which play key roles in regulating flowering time through photoperiod, vernalization, or functioning as an integrator of flowering signals.

Parasitic plants differ from free-living plants, as they have evolved a heterotrophic lifestyle, relying on haustoria that connect to the host’s vascular system to get resources for growth and development. Parasitic plants make up about 1% of angiosperms in the world, including about 4,500 species, representing at least 12 independent evolutionary events from autotrophs into parasitic plants (Westwood et al., 2010). Parasitic plants could be classified into hemiparasites and holoparasites, with the distinction being that the former can carry out partial or complete photosynthesis while the latter lacks the photosynthetic capacity. Some of these parasitic plants of Orobanchaceae, such as *Phtheirospermum*, *Striga*, are considered as agricultural weeds, which seriously jeopardize crops (Clarke et al., 2019; Kountche et al., 2019).

**FIGURE 1:**
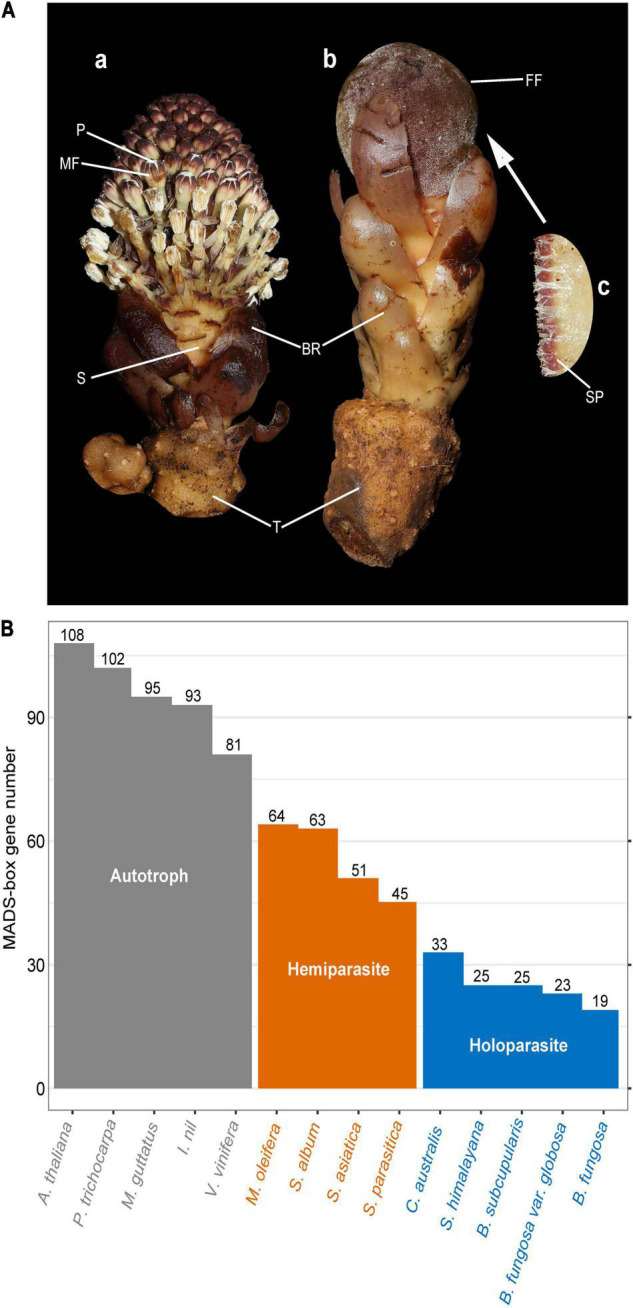
Morphological traits of *Balanophora* plants and MADS-box gene number. **(A)** Morphological structure of *Balanophora* plants (*B. fungosa* var. *globosa*). **(a,b)** Extremely simplified male plant and the female plant. **(c)** Female flower structure was observed under an electron microscope. FF, female flower; MF, male flower; S, stem; BR, bract; T, tuber; SP, spadicale; P, pollen. Image courtesy of Runxian Yu (CC BY-SA 2.0). **(B)** The number of MADS-box genes in *B. subcupularis*, *B. fungosa* var. *globosa*, and reference species. Fourteen species were selected according to the degree of parasitism and genetic relationship. Gray, orange, and blue indicate autotrophic plants, hemiparasite, and holoparasite, respectively. The bar chart shows the number of MADS-box genes in each species. The iTAK (http://itak.feilab.net/cgi-bin/itak/online_itak.cgi) and plantTFdb (http://planttfdb.gao-lab.org/prediction.php) programs were used to identify the MADS-box genes of these species.

Santalales is one of the largest family of parasitic plants, comprising autotrophic, hemiparasitic and holoparasitic plants (Chen et al., 2020), among which the holoparasitic plants of Balanophoraceae exhibit special morphological structure and extreme manifestation of parasitism. The aboveground part of the Balanophoraceae plants resembles the appearance of basidiomycetes fungi, consisting of a thickened and fleshy inflorescence, with or without scaly leaves (bracts), monoecious or dioecious (Shivamurthy et al., 1981a; Eberwein et al., 2009). Due to the holoparasitic habit, the vegetative and reproductive systems of Balanophoraceae plants are severely reduced, with the tuber serving as the only remaining vegetative organ from which the floral organs eventually emerge (Shivamurthy et al., 1981b). Unusually, 5–10 hypodermal cell-layers of the tuber re-differentiated into inflorescence meristem, which lacks a morphologically distinguishable epidermis and characteristically aligned hypodermal cell layers, indicating the special origin of flowers in *Balanophora* (Shivamurthy et al., 1981b). The female flowers of Balanophoraceae plants are very simple, filamentous structures, without tepals, only a style and a brief ovary, and a spadicale that overlays the surface of inflorescence (Figure 1A). Male flowers are also simple and consist of perianth and pollen (Figure 1A; Eberwein et al., 2009). Moreover, the tuber contains two vascular systems, one derived from the parasitic plant and the other is a complex tissue containing the parasitic and host plant tissue (Hsiao et al., 1994, 1995).

Previous studies indicated that there was a different degree of gene loss in hemiparasitic plant *Striga asiatica*, hemi-holoparasitic plant *Cuscuta australis* and holoparasitic plant *Sapria himalayana* according to the degree of host dependence, among which about 44% genes were lost in the genome of *Sapria* (Cai et al., 2021), we also found that gene redundancy was greatly reduced in two *Balanophora* species genomes, especially transcriptional factors, and extremely convergent gene loss was identified in two lineages of holoparasites, *Balanophora* and *Sapria himalayana* (Rafflesiaceae).

To explore the initiation, development and regulation of flowering in *Balanophora*, we identified the MADS-box genes in *B. subcupularis* (monoecious) and *B. fungosa* var. *globosa* (diocious). We analyzed the gene structures, conserved motifs, phylogeny, and tissue-specific expression of those genes. In addition, we also studied the gene loss pattern and further discussed the potential loss of the MADS-box genes related to the flowering regulation pathways in *B. subcupularis* and *B. fungosa* var. *globosa* and other holoparasitic plants. The results of our study can improve our understanding of the evolution and functions of MADS-box genes in *B. subcupularis* and *B. fungosa* var. *globosa* and would lay a foundation for further studies of the flowering development and regulation mechanisms in parasitic plants.

## Materials and Methods

### Identification of MADS-Box Genes in *Balanophora subcupularis, Balanophora fungosa* var. *globosa*, and Reference Species

In this study, we selected fourteen species based on the degree of parasitism, comprising seven orders (Supplementary Table 1), including holoparasitic *B. subcupularis*, *B. fungosa* var. *globosa* and *B. fungosa* (Santalales) (Leebens-Mack et al., 2019), *S. himalayana* (Malpighiales) (Cai et al., 2021), *C. australis* (Solanales) (Sun et al., 2018), hemiparasitic *S. asiatica* (Lamiales) (Yoshida et al., 2019), *S. parasitica* var. *graciliflora* (Santalales), *S. album* (Santalales) (Dasgupta et al., 2019), *M. oleifera* (Santalales) (Xu et al., 2019) and their close relatives. The genomes of *Balanophora* plants were obtained from China National GeneBank DataBase (CNGBdb) (CNP0003054). We employed iTAK^1^ and TFplantdb^2^ to search the MADS-box genes from genomes analyzed in this study. Then, all obtained MADS-box gene sequences of *B. subcupularis* and *B. fungosa* var. *globosa* were further analyzed in SMART,^3^ Pfam,^4^ CDD^5^ databases to verify the existence of MADS domain.

### Phylogenetic Analyses

A total of 829 MADS-box protein sequences from a total of 14 species and one MADS-box gene of *Aquilegia coerulea* as an outgroup were aligned using MAFFT software,^6^ and the phylogenetic tree was constructed by IQ-TREE^7^ program based on the maximum likelihood (ML) method. The parameters were set to −m GTR + R, −bb 1000, −alrt 1000, and the best alternative model is determined by model testing software. The tree was visualized by Evolview^8^ software.

### Gene Structure and Conserved Motif Analyses

According to the annotation information of *B. subcupularis* and *B. fungosa* var. *globosa*, the intron and exon composition of MADS-box gene were mapped by GSDS 2.0 (Gene Structure Display Server).^9^ The software MEME^10^ was used to search for the conservative motifs in the MADS-box genes of *Balanophora.* The parameter was as follows: the maximum number of motifs was 20, and the motifs width was set to 6–200 amino acids. The motifs were annotated by the Pfam database (see text footnote 4). The number of introns of species analyzed in this study was calculated according to the annotated information. The box plots of the intron length of *B. subcupularis*, *B. fungosa* var. *globosa*, and reference species were plotted using R packages.

### Confirmation of Gene Loss

We identified MADS-box gene loss primarily according to the phylogenetic tree at the ortholog level with *Arabidopsis thaliana* and *Vitis vinifera*. We divided the phylogenetic tree into ortholog groups and allowed the existence of paralogs of *Arabidopsis* in the same group, each ortholog group was assigned according to Supplementary Figure 2, and the primary identification of gene loss was identified based on it. For those absent genes in *Balanohpora*, orthologs from *Arabidopsis* and *V. vinifera* were used to predict against the assembly again (e-value threshold of 1e-5, coverage threshold of 0.25), the new annotations were checked manually including conserved domains, premature stop codons, and the transcriptome evidences. Then the manually confirmed gene models were used to construct a new phylogenetic tree, and gene loss was identified at the ortholog level again, and the absent genes in each orthologs group were considered as gene loss.

### Gene Loss Identification in Particular Pathways

For those important flowering regulation pathways, we further identified the gene loss through orthologs modified from Sun et al. (2018). First, BLASTP was used for all-to-all proteins alignment with e-value of 1e-5, after that, we employed OrthoFinder (v.2.3.7) to cluster genes with a Markov inflation index of 1.5 and a maximum e-value of 1e-5; next, the absent genes in parasitic plants will be searched against their genomes with homologs from *Arabidopsis* and *V. vinifera* (e-value threshold of 1e-5, coverage threshold of 0.25), and new annotated gene models will be included for the next step; then, phylogenetic trees were constructed with IQ-TREE in each orthogroup generated in the last step; each tree was divided into subgroups, and each subgroup must be included at least one *Arabidopsis* gene; finally, the gene numbers for every species in each subgroup were counted as the ortholog gene number.

### Expression Analysis of Flowering Related Genes in *Balanophora* and Host

The expression profiles of flowering regulate genes in different tissues during the development of *Balanophora* were explored using transcriptome data downloaded from CNGBdb (CNP0003054). The expression data included three stages, stage 1: samples containing different developmental stages, LC21-YT1-3 (tuber sizes < 8 mm, duplicate: LC21-YT1, LC21-YT2, and LC21-YT3), LC21-YT4-6 (tuber sizes: 8∼15 mm, duplicate: LC21-YT4, LC21-YT5, and LC21-YT6), stage 3 samples without visible inflorescence tissues, tuber and host root were collected and named as LC24-YT1-3 (duplicate: LC24-YT1, LC24-YT2, and LC24-YT3), stage 3 samples with grown inflorescence were separated into different tissues, including tuber LC24-T3-32 (duplicate: LC24-T3 and LC24-T32), male inflorescence LC22-MF1-3 (duplicate: LC22-MF1, LC22-MF2, LC22-MF3), female inflorescence LC24-FF1-3 (LC24-FF1, LC24-FF2, LC24-FF3), inflorescence stem LC24-S1-2 (duplicate: LC24-S1, LC24-S2), and bracts (LC23-BR1). The heatmap was generated by taking the average of these duplicates.

Because the tuber of *Balanophora* also contains tissues from the host, we carefully classified the reads into two distinct species to avoid possible contaminations. First, the gene set of the *Balanophora* and its host were decontaminated (removal of genes potentially from fungi, bacteria), and then combined. Next, bowtie2 was used to map the high-quality reads from each tissue with the combined gene models of *Balanophora* and its host. Those reads which could be mapped into both *Balanophora* and its host were removed. Finally, the mapped reads were divided into two species.

Using the Cufflinks pipeline^11^ the FPKM (Fragments Per Kilobase of transcript per Million mapped reads) value of genes in each tissue was calculated, and low-expressed and non-expressed genes were filtered out (FPKM < 10). There were three replicates for each tissue, and we took the average FPKM of the three replicates for the expression analysis. The expression levels were visualized using heatmap tools with FPKM data in each tissue in Hiplot.^12^ DESeq2 was used to calculate modest estimates of folding changes and dispersion of RNA-seq data, and log2 (fold change) >1, FDR < 0.05 was considered to be a differentially expressed gene.

## Results

### Dramatic Loss of MADS-Box Genes in Holoparasitic Plants

To study the evolution of MADS-box gene family in parasitic plants, we identified the MADS-box genes from parasitic plants and their autotrophic relatives (Figure 1B). Totally, 25 and 23 candidate genes were identified as MADS-box genes of *B. subcupularis* and *B. fungosa* var. *globosa*, respectively (Figure 1B). Then we named them BsubMADS1-25 and BgloMADS1-23 (Supplementary Table 2). Compared with *Arabidopsis* (108) and *Vitis* (81), we found that the gene number of this family in free-living plants was about twice that in hemiparasitic plants and three-five times that in holoparasitic plants, indicating that the number of MADS-box genes showed a stepwise loss trend with the increasing levels of parasitism. We found that the MADS-box gene family contracted sharply in holoparasitic *B. subcupularis*, *B. fungosa* var. *globosa*, *S. himalayana*, and their number of MADS-box genes is even fewer than *Amborella trichopoda* (36) (Albert et al., 2013). So, we inferred that the MADS-box genes of holoparasitic plants are drastically lost during the evolutionary process.

### Phylogenetic Analysis of MADS-Box Genes in *Balanophora subcupularis* and *Balanophora fungosa* var. *globosa*

**FIGURE 2:**
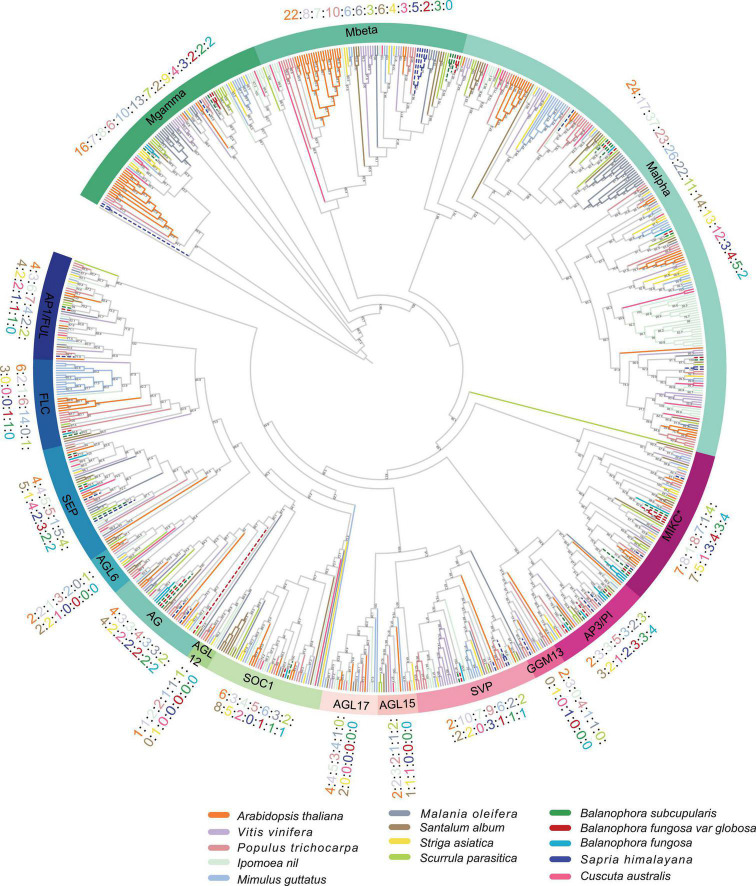
Phylogenetic tree of MADS-box genes in *B. subcupularis*, *B. fungosa* var. *globosa*, and reference species. The phylogenetic tree was constructed using the maximum likelihood (ML) method of IQ-TREE program (see text footnote 7). And visualized and decorated the tree by Evolview (see text footnote 8). Taking *Arabidopsis* as a reference, the tree was divided into 16 subfamilies (Smaczniak et al., 2012; Fatima et al., 2020). Different species are represented in different colors. Orange, green, blue, and red dotted lines represent *Arabidopsis*, *B. subcupularis*, *S. himalayana*, *B. fungosa* var. *globosa*, respectively. The numbers show the number of MADS-box genes of parasitic plants and reference species in each subfamily. The Bootstraps are ≥ 50. See Supplementary Figure 2 for more details. The alignment information of these genes are available in Supplementary Table 9.

To investigate the evolutionary relationship of MADS-box genes between parasitic plants, we constructed a phylogenetic tree that included autotrophic and parasitic plants (Figure 2). They were categorized into two types: type I (Mα, Mβ, and Mγ) and type II (MIKC^C^ and MIKC*) (Supplementary Table 3), which were further subdivided into sixteen subgroups following the previous classification (Smaczniak et al., 2012; Fatima et al., 2020). Based on the phylogenetic trees, ten and eight genes were classified as type I in *B. subcupularis* and *B. fungosa* var. *globosa*, respectively, and 15 genes were classified as type II in *B. subcupularis* and *B. fungosa* var. *globosa*, respectively. There are at least 21 MADS-box clades in the Most Recent Common Ancestor (MRCA) of extant angiosperms (Albert et al., 2013). While in our study we found that six clades were totally absent in *Balanophora* and seven clades were absent in *Sapria*, during which four clades were convergently lost (Supplementary Table 4).

We noticed several losses of whole subfamilies in *B. subcupularis* and *B. fungosa* var. *globosa*, such as the *AGL17*, *AGL15*, *AGL12*, *AGL6*, and *GNETUM GNEMON MADS13* (*GGM13*) clades, among which *AGL6/12/15/17* function in the flowering transition, and *TRANSPARENT TESTA16* (*TT16*) gene of GGM13 subfamily involved in seed pigmentation and embryo development (Erdmann et al., 2010). Most genes of AGL12 and AGL17 subfamilies are mainly involved in root development in addition to flowering (Tapia-López et al., 2008; Puig et al., 2013), which may be consistent with the lack of typical roots in *Balanophora*. It is worth noting that several genes classified in type II only contained the MADS domain confirmed by domain identification against Pfam database (Supplementary Figures 1, 2). Phylogenetic analysis showed that these non-K domain genes were more closely related to type II, suggesting that they may have lost their K domain during evolution. Further evidence in the following part such as intron number also supported the classification of these genes in type II.

### Modified Gene Structure and Conserved Motif in MADS-Box Genes of *Balanophora*

**FIGURE 3:**
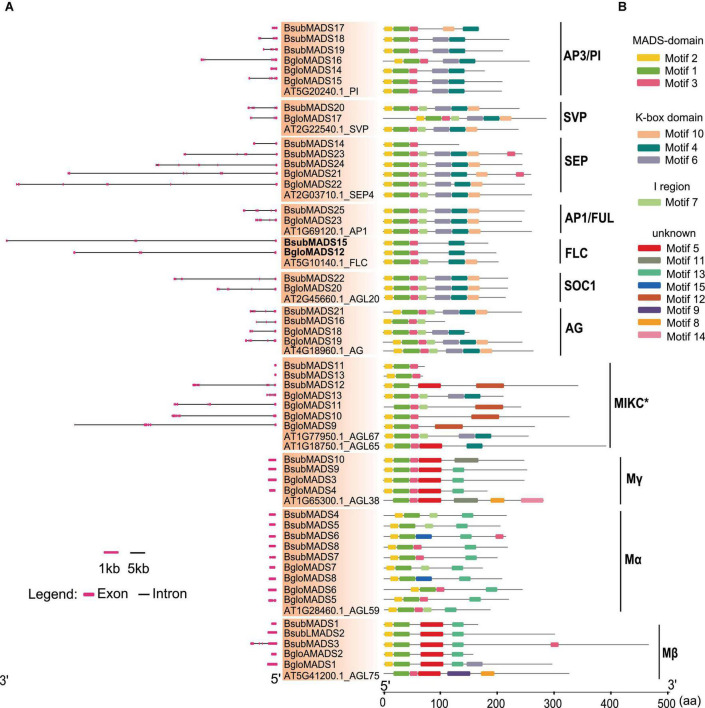
Gene structure and conserved motifs analysis of MADS-box genes in *B. subcupularis* and *B. fungosa* var. *globosa.* **(A)** The exon-intron structure of BsubMADS genes and BgloMADS genes were predicted by Gene Structure Display Server (GSDS) (see text footnote 9). The lines represent introns, and the red boxes indicate exons. The size of the introns and exons can be calculated using the bottom scale. **(B)** The motifs were identified by the Multiple EM for Motif Elicitation (MEME) program (http://meme-suite.org/tools/meme). Motifs 1–15 were represented by different colors, and the gene names and different groups are shown on the left side of the figure. The ruler at the bottom indicates the amino acid length of the sequences. The details of motifs are described in Supplementary Table 7.

To understand the structural diversity of MADS-box genes, we analyzed the intron and exon structure of these genes. We found that both *Balanophora* showed bimodal distribution between type I and II genes like other species (Wang et al., 2017, 2019; Fatima et al., 2020), significant differences in the number of introns were observed in the MADS-box genes of type I and type II in both *Balanophora* species (Figure 3). The number of the type II gene varied greatly, containing introns ranging from 0 to 8, among which 67% of the type II genes had at least five introns in both *Balanophora*. In addition, we also analyzed the number of introns of MADS-box genes of autotrophic plants and found that the intron number of type I genes was generally less than that of type II genes (Supplementary Table 5), suggesting that intron number in two types of the MADS-box gene family are conserved during evolution in flowering plants.

Intriguingly, there were very long introns inserted into the MADS-box genes of *Balanophora*, for example, the first intron of *FLC* homologue genes BsubMADS15 and BgloMADS12 are 64 kb and 56 kb, respectively, which are longer than other species (Supplementary Figure 3A). Moreover, the maximum intron length of MADS-box gene in *B. subcupularis*, *B. fungosa* var. *globosa*, and *S. himalayana* were longer than in other species (Supplementary Figure 3B). Furthermore, we analyzed the eight non-K domain genes of *B. subcupularis* and *B. fungosa* var. *globosa*, which were classified into type II on the phylogenetic tree. Previous studies showed that the length of the 1–6 exons of type II genes are conserved (Johansen et al., 2002). The average length of the first intron of the eight non-K domain genes in two *Balanophora* (197bp) was highly similar to that of type II (188bp), which was markedly smaller than that of type I genes (647bp) (Supplementary Table 6). Therefore, the results further proved the reliability of the classification of MADS-box non-K domain genes in the phylogenetic tree.

Next, we verified the conserved motifs of MADS-box genes in *B. subcupularis* and *B. fungosa* var. *globosa* using MEME program, then annotated the obtained motifs employing Pfam database. As a result, a total of 15 conserved motifs were identified and named motifs 1–15 (Supplementary Table 7). As shown in Figure 3, genes in the same family tend to have common patterns of conserved motifs, especially the type II gene whose domains are more conserved than type I, such as subgroups SOC1, SEP, *AP1/FUL*, *AP3/PI*. The MADS-box genes of *B. subcupularis* and *B. fungosa* var. *globosa* both contained motifs 1, 2, and 3, which were the most typical MADS domain according to Pfam database search. Motif 4, 6, and 10 were verified to be K-box domain, which was another conserved domain, and all MIKC^C^ type genes contained the K domain except BsubMADS14, BsubMADS16. We also observed that motif 7 represented the I domain that existed in all subgroups except for the subgroups *AP3/PI*, Mγ, Mβ of *B. subcupularis* and *B. fungosa* var. *globosa*. Besides, motif 8, 9, and 14 could be found in *Arabidopsis*, but absent in two *Balanophora.* Though the number and lengths of introns are variable in *Balanophora*, the motifs are conserved between *Balanophora* and *Arabidopsis*.

### Loss of Flowering Regulation Genes in Parasitic Plants

**FIGURE 4:**
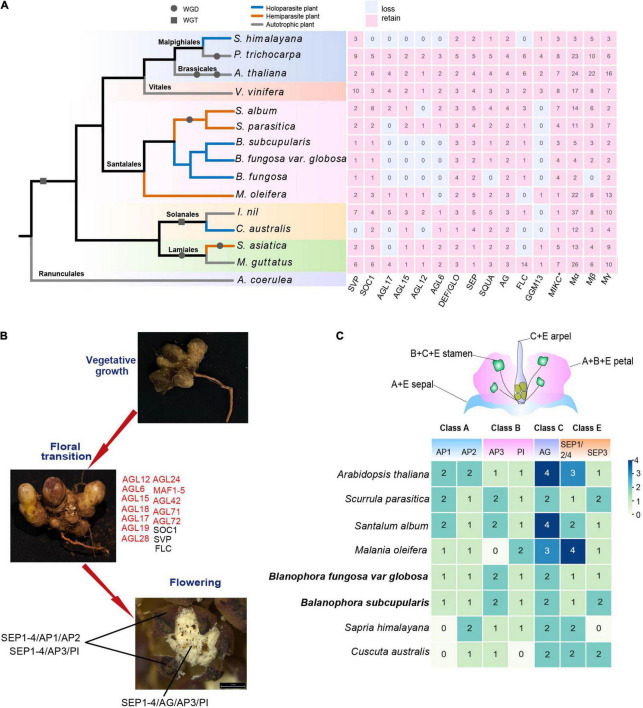
Gene loss of MADS-box genes in parasitic plants. **(A)** The gene loss and retention of 16 subgroups of MADS-box gene family in nine parasitic plants. The tree topology is modified from Nickrent (2020) with *Aquilegia coerulea* as outgroup, 14 species comprising seven orders (see Supplementary Table 1). The gray circle and square indicate WGD (whole genome duplication) and WGT (whole genome triplication) events, respectively, which were identified by 1KP (Leebens-Mack et al., 2019). The pink and white blocks indicate retain and loss of MADS-box genes in 16 subgroups, respectively. The green, blue, red branches represent autotrophic plants, hemiparasites, and holoparasites, respectively. **(B)** Gene loss of MADS-box genes in flowering transition. Genes marked in red represent loss and black represent retained genes in *B. subcupularis* and *B. fungosa* var. *globosa.* **(C)** A conventional flower structure of ABCE model genes. The numbers represent retained ABCE model genes in *B. subcupularis*, *B. fungosa* var. *globosa*, and reference species, while the numbers are obtained based on the phylogenetic tree.

The MADS-box gene family showed stepwise contraction from hemiparasitic to holoparasitic plants (Figure 1B). We analyzed the gene loss of nine parasitic plants, most MADS-box genes of parasitic plants showed a decreasing trend in each subfamily compared with autotrophic plants (Figure 4A). In addition, there was convergent genes loss among different lineages of parasitic plants, during which the most remarkable is the loss of subfamilies AGL6, AGL12, AGL15, AGL17 in *Balanophora* and *S. himalayana*, AGL12 and AGL17 were also lost in *C. australis*. There was some species-specific gene loss, such as the loss of *SVP* in *C. australis*, and the loss of *SOC1* in *S. himalayana* (Figure 4A). Hemiparasitic plants showed mild gene loss and most of them were also lost in holoparasites.

Based on the gene function of their homologies in *Arabidopsis*, we speculated that *AGL12* and *AGL17* genes may be related to the degradation of root and photoperiod regulation of flower transition in *Balanophora* and *S. himalayana* (Han et al., 2008; Tapia-López et al., 2008; Puig et al., 2013; Shu et al., 2020), they also convergently lost *AGL15* and *AGL6* genes, among which *AGL15* genes play roles in regulating flowering through photoperiod and somatic embryo development (Perry et al., 1999) and *AGL6* genes are involved in floral meristem regulation, development of floral organs, and ovule and seed, and *AGL6* genes also have possible roles in the development of male and female germline and gametophyte (Dreni and Zhang, 2016). Two *Balanophora* also lost *AGL63* and *TRANSPARENT TESTA16* (*TT16*) genes in GGM13 subfamily, which are involved in fruit development, seed formation, and embryo development (Erdmann et al., 2010). In conclusion, parasitic plants showed convergent and functional-biased gene loss in MADS-box gene families, which may be related to the parasitic lifestyle.

### Different Floral Meristem Gene Loss Patterns in Holoparasitic Plants

There are several genes related to floral meristem identity, including *AP1*, *FUL* (*AGL 8*), *CAL* (*CAULIFLOWER*), *AGL24*, *SVP*, *SOC1*, and *LEAFY* (*LFY*) (Gregis et al., 2009; Grandi et al., 2012). We observed that two *Balanophora* species only lost *FUL* gene, however, *AP1*/*CAL*, and *SOC1* genes were lost in *S. himalayana* (Supplementary Table 8). *FUL* gene promotes the identity of early flower meristem in coordination with *AP1* and appears to be partially redundant to the function of *AP1* (Ferrándiz et al., 2000). *AP1*, *AGL24*, and *SVP* control floral meristem identity redundantly by inhibiting the expression of class B, C, and E genes (Gregis et al., 2009). *SOC1* regulates the expression of *LFY*, and *LFY* is a non-MADS-box gene that links floral induction and floral development (Lee et al., 2008). These results showed that *Balanophora* and *S. himalayana* showed different levels of gene loss related to floral meristem, and they may lose those functional redundant genes which were unnecessary for them.

### Maintenance of ABCE Model Genes in Parasitic Plants

ABCE model genes coordinate together to determine floral organ identity (Soltis et al., 2007; Murai, 2013). Two *Balanophora* species retained all ABCE model genes (Figure 4C). While no *AP1* and no *SEP3* homologs were found in *S. himalayana*, suggesting that the A function does not rely on the function of *AP1* homologs or that the perianth has an entirely different contribution. Another holoparasite *C. australis* also lost *AP1* in class A, and *PI* in class B. The result suggested that holoparasites retained different levels of the ABCE genes, these may be a consequence of the highly modified floral structure or employing different flower identity genes in them.

### Dramatic Loss of Flowering Regulation Genes in Holoparasitic Plants

Flowering is essential to plant reproduction and can be regulated by a variety of pathways, such as photoperiod, circadian clock, vernalization, temperature, and autonomous, etc. (Liu et al., 2015). Previous studies have illustrated that there were at least 16 MADS-box genes involved in the regulation of flowering transition in *Arabidopsis*, but in our study, only three flowering signal integrators *SOC1*, *SVP*, *FLC* were found to be retained in *Balanophora*, and most genes were lost, for example, *AGL15*, *AGL17*, *AGL18*, *MAF1*, *MAF3/4/5* genes involved in photoperiod pathway (Adamczyk et al., 2007; Han et al., 2008; Kim and Sung, 2010), *AGL19* and *MAF2* genes associated with the vernalization pathway (Ratcliffe et al., 2003; Schönrock et al., 2006), *AGL28* gene involved in autonomic pathway (Yoo et al., 2006), the *SOC1*-like genes *AGL42*, *AGL71*, *AGL72* appeared to act through gibberellin-dependent pathway in *Arabidopsis* (Dorca-Fornell et al., 2011; Figure 4B and Supplementary Figure 4). We observed that the subfamilies AGL12, AGL15, AGL17, and AGL6 were convergently lost in *B. subcupularis*, *B. fungosa* var. *globosa*, and *S. himalayana*, while the subfamilies of SOC1 and FLC were specifically lost in *S. himalayana*, and GGM13 was specifically lost in *Balanophora* (Figure 4A). Among them, *SOC1* gene is an integrator of flowering signals involved in photoperiod, temperature, age, and gibberellin regulatory pathways in *Arabidopsis* to promote flowering transition, while *FLC* gene is a repressor of flowering, in part, it retards flowering by inhibiting the expression of *SOC1*. Another integrator *FLC* are involved in the vernalization and autonomic pathways (Michaels and Amasino, 1999, 2001; Michaels et al., 2005).

*Cuscuta australis* is another holoparasitic plant without roots and leaves that also lost many MADS-box genes. We observed that the subfamily SVP was specifically absent in *C. australis* (Figure 4A), which are involved in flowering time regulation in response to temperature changes by controlling the expression of the *FT* gene (Lee et al., 2007). Previous research has manifested that *C. australis* eavesdrops *FT* signals of the host to control flowering (Shen et al., 2020), taken together, we predicted that *Cuscuta* may have totally lost this SVP-FT signal pathway. In addition, it also lost subfamilies such as AGL17, AGL12, FLC, and GGM13 (Figure 4A), which showed convergent gene loss with holoparasitic plants. These indicated that holoparasites dramatically lost the flowering transition genes in MADS-box, *Balanophora* and *S. himalayana* showed a more severe loss.

Furthermore, we found that the gene loss in semi-parasitic plants with relatively intact body structures was not as pronounced as in holoparasitic plants. For example, *S. album* only lost subfamilies AGL12 and GGM13, *S. parasitica* lost AGL17 and GGM13 (Figure 4A). Loss of function in these subfamilies may be compensated by other genes, such as *AGL17*, a gene associated with root development, was functionally redundant with *AGL12* (Tapia-López et al., 2008; Puig et al., 2013).

We further explored the conserved subfamilies in *B. subcupularis* and *B. fungosa* var. *globosa* and discovered that the number of retained genes was decreased and only one gene was kept in some subgroups. For example, the subgroups SVP, SOC1, AP1/FUL, and FLC all retained only one gene in each *Balanophora* species, while there were more than two copies in *Arabidopsis* in each subgroup (Figure 4A). We concluded that the redundancy of MADS-box genes in holoparasitic plants was greatly reduced, and they would abandon sequence similar genes and retain the diversity of protein families.

**FIGURE 5:**
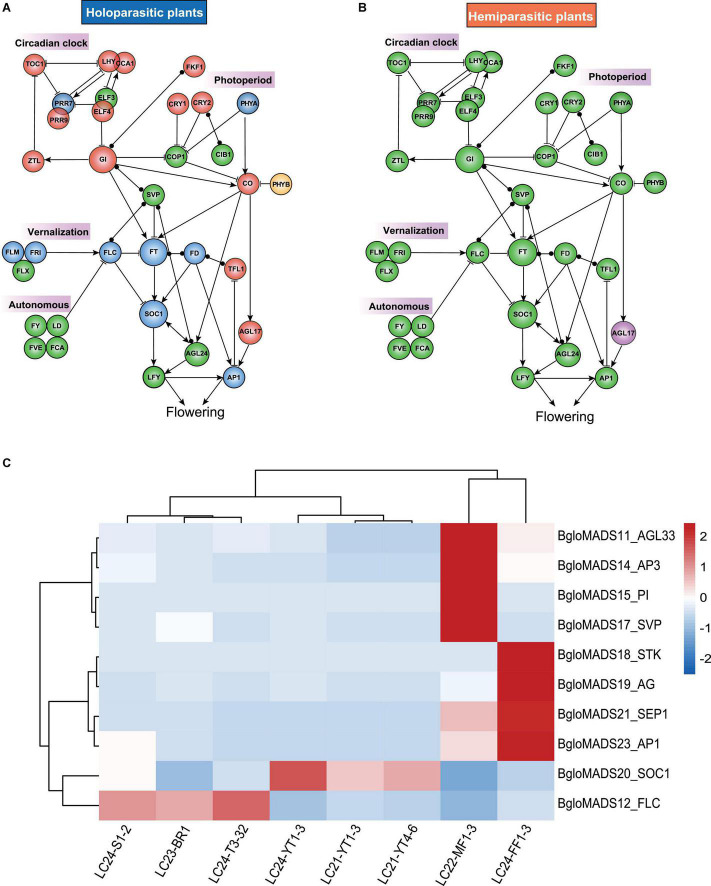
Four simplified pathways controlling flowering in parasitic plants and expression profiles of MADS-box genes for *Balanophora* (Photoperiod, Circadian clock, Autonomous, Vernalization Pathways). Panels **(A,B)** represent holoparasitic plants *B. subcupularis*, *B. fungosa* var. *globosa*, *Sapria* and hemiparasitic plants *S. parasitica*, *S. album*, *M. oleifera*, respectively. Red circles represent all lost in holoparasities. Green circles represent all retained in hemiparasites. Yellow represents only lost in *Balanophora*. Blue represents the loss in *Sapria*. Purple represents the loss in *S. parasitica*. The arrow indicates promoting gene activation, the blunt line indicates gene inhibition, and the two endpoints indicate no interaction in known directions. Genes *FT*, *LFY*, and *SOC1* integrate signals from multiple pathways. And in the pathways, *SVP*, *FLC*, *SOC1*, *AGL24*, *AGL17*, and *AP1* belong to MADS-box gene family, while the rest genes correspond to non-MADS-box genes. **(C)** The red and blue colors indicate the expression levels of BgloMADS genes from high to low, and white indicates the median expression level in the heatmap. The tissues cover three stages, stage 1: samples containing different tuber sizes, LC21-YT1-3 (<8 mm, duplicate: LC21-YT1, LC21-YT2, and LC21-YT3), LC21-YT4-6 (8∼15 mm, duplicate: LC21-YT4, LC21-YT5, and LC21-YT6), stage 2 samples without visible inflorescence tissues, tuber and host root were collected and named as LC24-YT1-3 (duplicate: LC24-YT1, LC24-YT2, and LC24-YT3), stage 3 samples with inflorescence were separated into different tissues, including tuber LC24-T3-32 (duplicate: LC24-T3 and LC24-T32), male inflorescence LC22-MF1-3 (duplicate: LC22-MF1, LC22-MF2, LC22-MF3), female inflorescence LC24-FF1-3 (LC24-FF1, LC24-FF2, LC24-FF3), inflorescence stem LC24-S1-2 (duplicate: LC24-S1, LC24-S2), and bracts (LC23-BR1).

We further investigated genes involved in flower regulation pathways, and found that two *Balanophora* plants and *S. himalayana* lost the circadian clock and photoperiod pathways (Figure 5A), comprising the important genes *CONSTANS* (*CO*), *TOC1*, *LHY*, *CCA1*, *EARLY FLOWERING 4* (*ELF4*), *Two-component response regulator-like APRR9* (*PRR9*) which are not MADS-box genes (Matsushika et al., 2000; Mizoguchi et al., 2002; McWatters et al., 2007; Nakamichi et al., 2010). However, *S. himalayana* also some species-specific loss, including *FT/FD*, *SOC1*, and *FLC* functioned as the floral integrator, the *FRIGIDA* (FRI) complex functioned in vernalization pathways (Choi et al., 2011; Figure 5A). Compared with holoparasitic plants, hemiparasitic plants kept all crucial flowering regulatory genes (Figure 5B). Based on these results, we inferred that *Balanophora* and *Sapria* may have lost the photoperiod and circadian clock pathways to control flowering.

### Conserved MADS-Box Genes Expression in *Balanophora*

To further confirm the functions of MADS-box genes in *Balanophora*, the transcriptome data from eight different tissues at different developmental stages were analyzed. In the MADS-box genes of *B. fungosa* var. *globosa*, the expression of eleven genes could be detected. The other genes cannot be identified because their expression levels were too low, or they have specific expression patterns, which were not collected in this study, it is also possible that they have lost their functions as they adapt to parasitic life. Several genes showed specific expression patterns (Figure 5C), in *B. fungosa* var. *globosa*, genes *AP1* (BgloMADS23), *SEP1* (BgloMADS21), *AG* (BgloMADS19), *STK* (BgloMADS18) were highly expressed in the female inflorescence, and *AG* (BgloMADS19) may be involved in the development of carpel, and *STK* (BgloMADS18) in controlling the development of ovules of the female flower. Furthermore, *PI* (BgloMADS15), *AP3* (BgloMADS14), *AGL33* (BgloMADS11), and *SVP* (BgloMADS17) were highly expressed in the male inflorescence, and *AG* (BgloMADS19) was also expressed in male flower, suggesting that *PI* (BgloMADS15), *AP3* (BgloMADS14), and *AG* (BgloMADS19) may be involved in the development of stamen. The expression pattern of these genes was consistent with the expression pattern of the ABCE model gene. In addition, we noticed that gene *FLC* (BgloMADS12) with extremely long introns showed high expression levels in the tuber and low expression levels in inflorescence (Figure 5C), which was similar to the expression pattern of *FLC* in *Arabidopsis* (highly expressed in the vegetative apex and root tissue). These findings revealed that the expression patterns of MADS-box genes in *B. fungosa* var. *globosa* were similar to other species, indicating that the function of these genes may be still conserved.

### Flower Regulatory Genes in the Host Are Upregulated in the Tuber

Because the vascular systems in the tuber of *Balanophora* contained both host and *Balanophora* tissues, the intimate connection may facilitate the usage of host signals, especially the long-distance mRNA movement between host and parasites (Kim et al., 2014). We investigated the expression levels of host genes related to regulating flowering in the tuber of *Balanophora*, and the results showed that several flowering regulation genes from the host were upregulated in the tuber compared to its own root, including (i) genes that can regulate *FT* or *FLC* levels transcriptionally or epigenetically, for example, AHL22, EBS, and bHLH63 function in the regulation of *FT* expression (Piñeiro et al., 2003; Yun et al., 2012; Liu et al., 2013); (ii) CO depression genes including MIP1A and CDF3 (Fornara et al., 2009; Graeff et al., 2016); (iii) Serine/threonine-protein kinase WNK1 regulates flowering time by modulating the photoperiod pathway (Wang et al., 2008; Supplementary Figure 5). Based on these lines of evidence, we speculated one possibility that *Balanophora* may take advantage of transcripts or proteins from the host plant to retain functionality in pathways of photoperiod regulation of flowering and has not lost associated regulatory pathways completely.

## Discussion

MADS-box transcription factors are important regulators, widely present in eukaryotes and highly conserved, and have been proven to play a crucial role in plant growth and development (Becker et al., 2000; Ng and Yanofsky, 2001; Parenicová et al., 2003). To the best of our knowledge, this is the first attempt to compare MADS-box genes among different parasitic plants. The number of MADS-box genes in holoparasitic plants was significantly reduced compared with that in autotrophic plants. Many MADS-box genes of parasitic plants have been lost during the process of adaption to parasitism.

### The Ongoing Loss of the K Domain in MIKC Subgroup Genes

A phylogenetic tree of the fourteen species was built and divided into sixteen subgroups based on the taxonomy of *Arabidopsis* (Smaczniak et al., 2012). Phylogenetic analysis revealed that there were eight non-K domain genes clustered into type II. This phenomenon was also found in MADS-box genes of rice, apple, and pear (Arora et al., 2007; Tian et al., 2015; Wang et al., 2017), indicating that it might be common in angiosperms. Studies have shown that MIKC genes are conserved in the length of the 1–6 exons (Johansen et al., 2002), so we analyzed the length of the first exon of these eight genes and found that the length was closer to that of type II genes but different from type I genes, among them, BgloMADS11 was highly expressed in inflorescence, indicated that this gene might be functional. This result illustrated that some MADS-box genes are probably experiencing K domain loss, and at least several genes without K domain are still functional.

### Severe Loss of Flowering-Related Genes and Peculiar Flowering Regulation Pattern in Holoparasitic Plants

Reports on the evolutionary mechanism of phenotypic adaptation indicated gene loss can be beneficial by providing an evolutionary mechanism for phenotypic adaptation (Albalat and Cañestro, 2016; Sahu et al., 2019; Wang et al., 2021; Li et al., 2022). Studies on the flowering mechanism of *C. australis* show that in order to ensure flowering, dodder eavesdropped on the flowering signal of the host and kept the synchronization of flowering (Sun et al., 2018; Shen et al., 2020). In this study, severe gene loss of flowering regulation genes was also found in *B. subcupularis*, *B. fungosa* var. *globosa*, and *S. himalayana*, especially the circadian clock and photoperiodic flowering pathways, but there were several photoperiodic flowering response genes from the host plant are upregulated in the chimeric tuber, speculating that *Balanophora* may take advantage of transcripts or proteins from the host to retain functionality in pathways of photoperiod regulation of flowering, instead of losing the regulatory pathway completely. It is probably a convergent strategy for parasitic plants to steal flowering signals from the host (Shen et al., 2020). However, in hemiparasitic plants, almost all the flowering regulation pathways were retained. Dramatic loss of flower regulation genes in holoparasites analyzed in this study indicated reduced robustness in parasites with extremely degraded structure and high levels of dependence on the host.

### Reduction of the MADS-Box Genes Redundancy in the Holoparasitic Plants

Redundant genes generally have a genetic compensation function (i.e., the loss of one gene can be compensated by another with overlapping functions and expression patterns), and probably establish a genetically robust system for adaption (Wagner, 2000; Dean et al., 2008; Rutter et al., 2017; Yang et al., 2022). Studies have indicated that the reduction of gene expression after replication promotes the long-term maintenance of duplicate genes and functional redundancy (Qian et al., 2010). Several subgroups showed genetic redundancy in MADS-box, for example, the *AP1/FUL* subgroup, including *AP1*/*CAL*, *FUL*, and *AGL79*, among which *AP1*/*CAL* and *FUL* played important roles in the transition to floral meristem, floral meristem development, perianth, or fruit development (Gu et al., 1998; Ferrándiz et al., 2000; Acri-Nunes-Miranda and Mondragón Palomino, 2014), only one member could be identified in *Balanophora* (retained *AP1*/*CAL*) and *Sapria* (retained *AGL79*), respectively (Figure 2). In FLC and SOC1 subgroups, six members were confined to *Arabidopsis*, respectively, and only one member in each subgroup was identified in *Balanophora*, with a complete absence in *Sapria*. The reduction of redundancy in holoparasites, together with the loss of other regulatory genes, indicated that holoparasites may employ a relatively simpler flowering regulation system compared to autotrophs. The simplicity may be due to the utilization of signals directly from the host, or the highly modified structure, or the reduced demand for additional robustness when hosts offer stable environments.

## Conclusion

Identification of MADS-box gene family in parasitic plants showed that the MADS-box genes family was contracted step by step from hemiparasites to holoparasites compared to autotrophs. Plenty of MADS-box genes may have been lost in *Balanophora* and *Sapria* genomes, and, they seem to have reduced redundancy in conserved subfamilies. Gene loss and redundancy reduction in *Balanophora* and *Sapria* indicated that they may not require a complicated network to regulate flowering due to their degraded structure, or relatively stable environments supported by hosts, or they directly use the flowering signals from the host. Overall, this is the first genome-wide analysis of the MADS-box gene family in hemi- and holo-parasites, and it contributes to our understanding of the MADS-box gene family’s categorization, gene loss pattern, and flowering regulatory mechanism in parasitic plants.

## Data Availability Statement

The datasets generated for this study can be found in the Supplementary Material, and China National GeneBank DataBase (CNGBdb) database under the accession number: CNP0003054.

## Author Contributions

HL, XC, and SS led and designed this project. KD, HF, DF, and WZ analyzed the data. KD wrote the original draft manuscript. XC, SS, and KW revised the manuscript. All authors contributed to the article and approved the submitted version.

## Conflict of Interest

The authors declare that the research was conducted in the absence of any commercial or financial relationships that could be construed as a potential conflict of interest.

## Publisher’s Note

All claims expressed in this article are solely those of the authors and do not necessarily represent those of their affiliated organizations, or those of the publisher, the editors and the reviewers. Any product that may be evaluated in this article, or claim that may be made by its manufacturer, is not guaranteed or endorsed by the publisher.

## Footnotes

1
http://itak.feilab.net/cgi-bin/itak/online_itak.cgi

2
http://planttfdb.gao-lab.org/prediction.php

3
http://smart.embl.de/

4
http://pfam.xfam.org/

5
https://www.ncbi.nlm.nih.gov/Structure/bwrpsb/bwrpsb.cgi

6
https://www.ebi.ac.uk/Tools/msa/mafft/

7
http://www.iqtree.org/

8
https://www.evolgenius.info/evolview

9
http://gsds.cbi.pku.edu.cn

10
http://meme-suite.org

11
http://cufflinks.cbcb.umd.edu/

12
https://hiplot.com.cn/basic/heatmap

## References

[B1] Acri-Nunes-Miranda R. Mondragón Palomino M. (2014). Expression of paralogous SEP-, FUL-, AG- and STK-like MADS-box genes in wild-type and peloric *Phalaenopsis* flowers. *Front. Plant Sci*. 5:76. 10.3389/fpls.2014.00076 24659990 PMC3950491

[B2] Adamczyk B. J. Fernandez D. E. (2009). MIKC* MADS Domain Heterodimers Are Required for Pollen Maturation and Tube Growth in *Arabidopsis*. *Plant Physiol.* 149 1713–1723. 10.1104/pp.109.135806 19211705 PMC2663741

[B3] Adamczyk B. J. Lehti-Shiu M. D. Fernandez D. E. (2007). The MADS domain factors AGL15 and AGL18 act redundantly as repressors of the floral transition in *Arabidopsis*. *Plant J.* 50 1007–1019. 10.1111/j.1365-313X.2007.03105.x 17521410

[B4] Albalat R. Cañestro C. (2016). Evolution by gene loss. *Nat. Rev. Genet*. 17 379–391. 10.1038/nrg.2016.39 27087500

[B5] Albert V. A. Barbazuk W. B. dePamphilis C. W. Der J. P. Leebens-Mack J. Ma H. (2013). The *Amborella* Genome and the Evolution of Flowering Plants. *Science* 342:1241089. 10.1126/science.1241089 24357323

[B6] Alvarez-Buylla E. R. Pelaz S. Liljegren S. J. Gold S. E. Burgeff C. Ditta G. S. (2000). An ancestral MADS-box gene duplication occurred before the divergence of plants and animals. *Proc. Natl. Acad. Sci. U S A*. 97 5328–5333. 10.1073/pnas.97.10.5328 10805792 PMC25828

[B7] Arora R. Agarwal P. Ray S. Singh A. K. Singh V. P. Tyagi A. K. (2007). MADS-box gene family in rice: genome-wide identification, organization and expression profiling during reproductive development and stress. *BMC Genom.* 8:242. 10.1186/1471-2164-8-242 17640358 PMC1947970

[B8] Becker A. Winter K.-U. Meyer B. Saedler H. Theißen G. (2000). MADS-Box Gene Diversity in Seed Plants 300 Million Years Ago. *Mol. Biol. Evol.* 17 1425–1434. 10.1093/oxfordjournals.molbev.a026243 11018150

[B9] Cai L. Arnold B. J. Xi Z. Khost D. E. Patel N. Hartmann C. B. (2021). Deeply Altered Genome Architecture in the Endoparasitic Flowering Plant *Sapria himalayana* Griff. (Rafflesiaceae). *Curr. Biol.* 31 1002–1011.e9. 10.1016/j.cub.2020.12.045 33485466

[B10] Chen X. Fang D. Wu C. Liu B. Liu Y. Sahu S. K. (2020). Comparative plastome analysis of root-and stem-feeding parasites of Santalales untangle the footprints of feeding mode and lifestyle transitions. *Genom. Biol. Evol.* 12 3663–3676. 10.1093/gbe/evz271 31845987 PMC6953812

[B11] Choi K. Kim J. Hwang H. J. Kim S. Park C. Kim S. Y. (2011). The FRIGIDA complex activates transcription of FLC, a strong flowering repressor in *Arabidopsis*, by recruiting chromatin modification factors. *Plant Cell* 23 289–303. 10.1105/tpc.110.075911 21282526 PMC3051252

[B12] Clarke C. R. Timko M. P. Yoder J. I. Axtell M. J. Westwood J. H. (2019). Molecular Dialog Between Parasitic Plants and Their Hosts. *Annu. Rev. Phytopathol.* 57 279–299. 10.1146/annurev-phyto-082718-100043 31226021

[B13] Dasgupta M. G. Ulaganathan K. Dev S. A. Balakrishnan S. (2019). Draft genome of *Santalum album* L. provides genomic resources for accelerated trait improvement. *Tree Genet*. *Genomes* 15:34. 10.1007/s11295-019-1334-9

[B14] Dean E. J. Davis J. C. Davis R. W. Petrov D. A. (2008). Pervasive and persistent redundancy among duplicated genes in Yeast. *PLoS Genet.* 4:e1000113. 10.1371/journal.pgen.1000113 18604285 PMC2440806

[B15] Dorca-Fornell C. Gregis V. Grandi V. Coupland G. Colombo L. Kater M. M. (2011). The *Arabidopsis* SOC1-like genes AGL42, AGL71 and AGL72 promote flowering in the shoot apical and axillary meristems. *Plant J.* 67 1006–1017. 10.1111/j.1365-313X.2011.04653.x 21609362

[B16] Dreni L. Zhang D. (2016). Flower development: the evolutionary history and functions of the AGL6 subfamily MADS-box genes. *J. Exp. Bot.* 67 1625–1638. 10.1093/jxb/erw046 26956504

[B17] Eberwein R. Nickrent D. Weber A. (2009). Development and morphology of flowers and inflorescences in *Balanophora Papuana* And *B. Elongata* (Balanophoraceae). *Am. J. Bot.* 96 1055–1067. 10.3732/ajb.0800289 21628256

[B18] Ehlers K. Bhide A. S. Tekleyohans D. G. Wittkop B. Snowdon R. J. Becker A. (2016). The MADS Box Genes ABS, SHP1, and SHP2 Are Essential for the Coordination of Cell Divisions in Ovule and Seed Coat Development and for Endosperm Formation in *Arabidopsis thaliana*. *PLoS One* 11:e0165075. 10.1371/journal.pone.0165075 27776173 PMC5077141

[B19] Erdmann R. Gramzow L. Melzer R. Theißen G. Becker A. (2010). GORDITA (AGL63) is a young paralog of the *Arabidopsis thaliana* Bsister MADS box gene ABS (TT16) that has undergone neofunctionalization. *Plant J*. 63 914–924. 10.1111/j.1365-313X.2010.04290.x 20598091

[B20] Fatima M. Zhang X. Lin J. Zhou P. Zhou D. Ming R. (2020). Expression profiling of MADS-box gene family revealed its role in vegetative development and stem ripening in *S. spontaneum*. *Sci. Rep.* 10:20536. 10.1038/s41598-020-77375-6 33239664 PMC7688973

[B21] Ferrándiz C. Gu Q. Martienssen R. Yanofsky M. F. (2000). Redundant regulation of meristem identity and plant architecture by FRUITFULL, APETALA1 and CAULIFLOWER. *Development* 127 725–734. 10.1242/dev.127.4.725 10648231

[B22] Fornara F. Panigrahi K. C. Gissot L. Sauerbrunn N. Rühl M. Jarillo J. A. (2009). *Arabidopsis* DOF transcription factors act redundantly to reduce CONSTANS expression and are essential for a photoperiodic flowering response. *Dev. Cell* 17 75–86. 10.1016/j.devcel.2009.06.015 19619493

[B23] Garay-Arroyo A. Ortiz-Moreno E. de la Paz Sánchez M. Murphy A. S. García-Ponce B. Marsch-Martínez N. (2013). The MADS transcription factor XAL2/AGL14 modulates auxin transport during *Arabidopsis* root development by regulating PIN expression. *EMBO J*. 32 2884–2895. 10.1038/emboj.2013.216 24121311 PMC3817466

[B24] Graeff M. Straub D. Eguen T. Dolde U. Rodrigues V. Brandt R. (2016). MicroProtein-Mediated Recruitment of CONSTANS into a TOPLESS Trimeric Complex Represses Flowering in *Arabidopsis*. *PLoS Genet.* 12:e1005959. 10.1371/journal.pgen.1005959 27015278 PMC4807768

[B25] Gramzow L. Ritz M. S. Theissen G. (2010). On the origin of MADS-domain transcription factors. *Trends Genet.* 26 149–153. 10.1016/j.tig.2010.01.004 20219261

[B26] Grandi V. Gregis V. Kater M. M. (2012). Uncovering genetic and molecular interactions among floral meristem identity genes in *Arabidopsis thaliana*. *Plant J*. 69 881–893. 10.1111/j.1365-313X.2011.04840.x 22040363

[B27] Gregis V. Sessa A. Dorca-Fornell C. Kater M. M. (2009). The *Arabidopsis* floral meristem identity genes AP1, AGL24 and SVP directly repress class B and C floral homeotic genes. *Plant J*. 60 626–637. 10.1111/j.1365-313X.2009.03985.x 19656343

[B28] Gu Q. Ferrandiz C. Yanofsky M. F. Martienssen R. (1998). The FRUITFULL MADS-box gene mediates cell differentiation during *Arabidopsis* fruit development. *Development* 125 1509–1517. 10.1242/dev.125.8.1509 9502732

[B29] Han P. García-Ponce B. Fonseca-Salazar G. Alvarez-Buylla E. R. Yu H. (2008). AGAMOUS-LIKE 17, a novel flowering promoter, acts in a FT-independent photoperiod pathway. *Plant J.* 55 253–265. 10.1111/j.1365-313X.2008.03499.x 18363787

[B30] Hartmann U. Höhmann S. Nettesheim K. Wisman E. Saedler H. Huijser P. (2000). Molecular cloning of SVP: a negative regulator of the floral transition in *Arabidopsis*. *Plant J.* 21 351–360. 10.1046/j.1365-313x.2000.00682.x 10758486

[B31] Henschel K. Kofuji R. Hasebe M. Saedler H. Münster T. Theissen G. (2002). Two ancient classes of MIKC-type MADS-box genes are present in the moss *Physcomitrella patens*. *Mol. Biol. Evol.* 19 801–814. 10.1093/oxfordjournals.molbev.a004137 12032236

[B32] Hsiao S.-C. Mauseth J. D. Gomez L. D. (1994). Growth and Anatomy of the Vegetative Body of the Parasitic Angiosperm *Langsdorffia hypogaea* (Balanophoraceae). *Bull. Torrey Bot. Club.* 121 24–39. 10.2307/2996881

[B33] Hsiao S.-C. Mauseth J. D. Peng C.-I. (1995). Composite bundles, the host/parasite interface in the holoparasitic angiosperms *Langsdorffia and Balanophora* (Balanophoraceae). *Am. J. Bot.* 82 81–91. 10.1002/j.1537-2197.1995.tb15652.x

[B34] Johansen B. Pedersen L. B. Skipper M. Frederiksen S. (2002). MADS-box gene evolution-structure and transcription patterns. *Mol. Phylogenet. Evol.* 23 458–480. 10.1016/s1055-7903(02)00032-5 12099799

[B35] Kaufmann K. Melzer R. Theissen G. (2005). MIKC-type MADS-domain proteins: structural modularity, protein interactions and network evolution in land plants. *Gene* 347 183–198. 10.1016/j.gene.2004.12.014 15777618

[B36] Kim D. H. Sung S. (2010). The Plant Homeo Domain finger protein, VIN3-LIKE 2, is necessary for photoperiod-mediated epigenetic regulation of the floral repressor, MAF5. *Proc. Natl. Acad. Sci. U S A.* 107 17029–17034. 10.1073/pnas.1010834107 20837520 PMC2947895

[B37] Kim G. LeBlanc M. L. Wafula E. K. dePamphilis C. W. Westwood J. H. (2014). Genomic-scale exchange of mRNA between a parasitic plant and its hosts. *Science* 345 808–811. 10.1126/science.1253122 25124438

[B38] Kountche B. A. Jamil M. Yonli D. Nikiema M. P. Blanco-Ania D. Asami T. (2019). Suicidal germination as a control strategy for *Striga hermonthica* (Benth.) in smallholder farms of sub-Saharan Africa. *Plants People Planet* 1 107–118. 10.1002/ppp3.32

[B39] Lee J. Oh M. Park H. Lee I. (2008). SOC1 translocated to the nucleus by interaction with AGL24 directly regulates leafy. *Plant J.* 55 832–843. 10.1111/j.1365-313X.2008.03552.x 18466303

[B40] Lee J. H. Yoo S. J. Park S. H. Hwang I. Lee J. S. Ahn J. H. (2007). Role of SVP in the control of flowering time by ambient temperature in *Arabidopsis*. *Genes Dev.* 21 397–402. 10.1101/gad.1518407 17322399 PMC1804328

[B41] Leebens-Mack J. H. Barker M. S. Carpenter E. J. Deyholos M. K. Gitzendanner M. A. Graham S. W. (2019). One thousand plant transcriptomes and the phylogenomics of green plants. *Nature* 574 679–685. 10.1038/s41586-019-1693-2 31645766 PMC6872490

[B42] Li L. Chen X. Fang D. Dong S. Guo X. Li N. (2022). Genomes shed light on the evolution of *Begonia*, a mega-diverse genus. *New Phytol.* 234 295–310. 10.1111/nph.17949 34997964 PMC7612470

[B43] Liljegren S. J. Ditta G. S. Eshed Y. Savidge B. Bowman J. L. Yanofsky M. F. (2000). SHATTERPROOF MADS-box genes control seed dispersal in *Arabidopsis*. *Nature* 404 766–770. 10.1038/35008089 10783890

[B44] Liu J. Fu X. Dong Y. Lu J. Ren M. Zhou N. (2018). MIKC^C^-type MADS-box genes in *Rosa chinensis*: the remarkable expansion of ABCDE model genes and their roles in floral organogenesis. *Horticult. Res.* 5:25. 10.1038/s41438-018-0031-4 29736250 PMC5928068

[B45] Liu Y. Li X. Li K. Liu H. Lin C. (2013). Multiple bHLH proteins form heterodimers to mediate CRY2-dependent regulation of flowering-time in *Arabidopsis*. *PLoS Genet.* 9:e1003861. 10.1371/journal.pgen.1003861 24130508 PMC3794922

[B46] Liu Y. Yang J. Yang M. (2015). [Pathways of flowering regulation in plants]. *Sheng Wu Gong Cheng Xue Bao* 31 1553–1566. 26939439

[B47] Matsushika A. Makino S. Kojima M. Mizuno T. (2000). Circadian waves of expression of the APRR1/TOC1 family of pseudo-response regulators in *Arabidopsis thaliana*: insight into the plant circadian clock. *Plant Cell Physiol.* 41 1002–1012. 10.1093/pcp/pcd043 11100772

[B48] McWatters H. G. Kolmos E. Hall A. Doyle M. R. Amasino R. M. Gyula P. (2007). ELF4 is required for oscillatory properties of the circadian clock. *Plant Physiol.* 144 391–401. 10.1104/pp.107.096206 17384164 PMC1913775

[B49] Melzer R. Theissen G. (2009). Reconstitution of ‘floral quartets’ in vitro involving class B and class E floral homeotic proteins. *Nucleic Acids Res.* 37 2723–2736. 10.1093/nar/gkp129 19276203 PMC2677882

[B50] Michaels S. D. Amasino R. M. (1999). FLOWERING LOCUS C Encodes a Novel MADS Domain Protein That Acts as a Repressor of Flowering. *Plant Cell* 11 949–956. 10.1105/tpc.11.5.949 10330478 PMC144226

[B51] Michaels S. D. Amasino R. M. (2001). Loss of FLOWERING LOCUS C activity eliminates the late-flowering phenotype of FRIGIDA and autonomous pathway mutations but not responsiveness to vernalization. *Plant Cell* 13 935–941. 10.1105/tpc.13.4.935 11283346 PMC135534

[B52] Michaels S. D. Himelblau E. Kim S. Y. Schomburg F. M. Amasino R. M. (2005). Integration of Flowering Signals in Winter-Annual *Arabidopsis*. *Plant Physiol*. 137 149–156. 10.1104/pp.104.052811 15618421 PMC548846

[B53] Mizoguchi T. Wheatley K. Hanzawa Y. Wright L. Mizoguchi M. Song H. R. (2002). LHY and CCA1 are partially redundant genes required to maintain circadian rhythms in *Arabidopsis*. *Dev. Cell* 2 629–641. 10.1016/s1534-5807(02)00170-3 12015970

[B54] Moreno-Risueno M. A. Norman J. M. V. Moreno A. Zhang J. Ahnert S. E. Benfey P. N. (2010). Oscillating Gene Expression Determines Competence for Periodic *Arabidopsis* Root Branching. *Science* 329 1306–1311. 10.1126/science.1191937 20829477 PMC2976612

[B55] Murai K. (2013). Homeotic Genes and the ABCDE Model for Floral Organ Formation in Wheat. *Plants* 2 379–395. 10.3390/plants2030379 27137382 PMC4844379

[B56] Nakamichi N. Kiba T. Henriques R. Mizuno T. Chua N. H. Sakakibara H. (2010). PSEUDO-RESPONSE REGULATORS 9, 7, and 5 are transcriptional repressors in the *Arabidopsis* circadian clock. *Plant Cell* 22 594–605. 10.1105/tpc.109.072892 20233950 PMC2861452

[B57] Ng M. Yanofsky M. F. (2001). Function and evolution of the plant MADS-box gene family. *Nat. Rev. Genet.* 2 186–195. 10.1038/35056041 11256070

[B58] Nickrent D. L. (2020). Parasitic angiosperms: how often and how many? *Taxon* 69 5–27. 10.1002/tax.12195

[B59] Pan Z. J. Chen Y. Y. Du J. S. Chen Y. Y. Chung M. C. Tsai W. C. (2014). Flower development of *Phalaenopsis* orchid involves functionally divergent SEPALLATA-like genes. *New Phytol.* 202 1024–1042. 10.1111/nph.12723 24571782 PMC4288972

[B60] Parenicová L. de Folter S. Kieffer M. Horner D. S. Favalli C. Busscher J. (2003). Molecular and phylogenetic analyses of the complete MADS-box transcription factor family in *Arabidopsis*: new openings to the MADS world. *Plant Cell* 15 1538–1551. 10.1105/tpc.011544 12837945 PMC165399

[B61] Perry S. E. Lehti M. D. Fernandez D. E. (1999). The MADS-domain protein AGAMOUS-like 15 accumulates in embryonic tissues with diverse origins. *Plant Physiol.* 120 121–130. 10.1104/pp.120.1.121 10318690 PMC59244

[B62] Piñeiro M. Gómez-Mena C. Schaffer R. Martínez-Zapater J. M. Coupland G. (2003). EARLY BOLTING IN SHORT DAYS is related to chromatin remodeling factors and regulates flowering in *Arabidopsis* by repressing FT. *Plant Cell* 15 1552–1562. 10.1105/tpc.012153 12837946 PMC165400

[B63] Puig J. Meynard D. Khong G. N. Pauluzzi G. Guiderdoni E. Gantet P. (2013). Analysis of the expression of the AGL17-like clade of MADS-box transcription factors in rice. *Gene. Expr. Patterns* 13 160–170. 10.1016/j.gep.2013.02.004 23466806

[B64] Qian W. Liao B.-Y. Chang A. Y.-F. Zhang J. (2010). Maintenance of duplicate genes and their functional redundancy by reduced expression. *Trends Genet.* 26 425–430. 10.1016/j.tig.2010.07.002 20708291 PMC2942974

[B65] Ratcliffe O. J. Kumimoto R. W. Wong B. J. Riechmann J. L. (2003). Analysis of the *Arabidopsis* MADS AFFECTING FLOWERING gene family: MAF2 prevents vernalization by short periods of cold. *Plant Cell* 15 1159–1169. 10.1105/tpc.009506 12724541 PMC153723

[B66] Riechmann J. L. Meyerowitz E. M. (1997). Determination of floral organ identity by *Arabidopsis* MADS domain homeotic proteins AP1, AP3, PI, and AG is independent of their DNA-binding specificity. *Mol. Bio. Cell* 8 1243–1259. 10.1091/mbc.8.7.1243 9243505 PMC276150

[B67] Riechmann J. L. Wang M. Meyerowitz E. M. (1996). DNA-binding properties of *Arabidopsis* MADS domain homeotic proteins APETALA1, APETALA3, PISTILLATA and AGAMOUS. *Nucleic Acids Res.* 24 3134–3141. 10.1093/nar/24.16.3134 8774892 PMC146081

[B68] Rutter M. T. Wieckowski Y. M. Murren C. J. Strand A. E. (2017). Fitness effects of mutation: testing genetic redundancy in *Arabidopsis thaliana*. *J. Evol. Biol.* 30 1124–1135. 10.1111/jeb.13081 28387971

[B69] Sahu S. K. Liu M. Yssel A. Kariba R. Muthemba S. Jiang S. (2019). Draft Genomes of Two Artocarpus Plants, Jackfruit (*A. heterophyllus*) and Breadfruit (*A. altilis*). *Genes* 11:27. 10.3390/genes11010027 31878322 PMC7017358

[B70] Samach A. Onouchi H. Gold S. E. Ditta G. S. Schwarz-Sommer Z. Yanofsky M. F. (2000). Distinct roles of CONSTANS target genes in reproductive development of *Arabidopsis*. *Science* 288 1613–1616. 10.1126/science.288.5471.1613 10834834

[B71] Schilling S. Pan S. Kennedy A. Melzer R. (2018). MADS-box genes and crop domestication: the jack of all traits. *J. Exp. Bot.* 69 1447–1469. 10.1093/jxb/erx479 29474735

[B72] Schönrock N. Bouveret R. Leroy O. Borghi L. Köhler C. Gruissem W. (2006). Polycomb-group proteins repress the floral activator AGL19 in the FLC-independent vernalization pathway. *Genes Dev.* 20 1667–1678. 10.1101/gad.377206 16778081 PMC1482485

[B73] Shen G. Liu N. Zhang J. Xu Y. Baldwin I. T. Wu J. (2020). *Cuscuta australis* (dodder) parasite eavesdrops on the host plants’ FT signals to flower. *Proc. Natl. Acad. Sci. U S A.* 117 23125–23130. 10.1073/pnas.2009445117 32868415 PMC7502711

[B74] Shivamurthy G. R. Arekal G. D. Swamy B. G. L. (1981a). Establishment, Structure and Morphology of the Tuber of *Balanophora*. *Ann. Bot.* 47 735–745. 10.1093/oxfordjournals.aob.a086072

[B75] Shivamurthy G. R. Swamy B. G. L. Arekal G. D. (1981b). Ontogeny and Organization of the Inflorescence in *Balanophora*. *Ann. Bot.* 48 853–859. 10.1093/oxfordjournals.aob.a086192

[B76] Shu J. Chen C. Kohalmi S. E. Cui Y. (2020). Evidence that AGL17 is a significant downstream target of CLF in floral transition control. *Plant Signal Behav.* 15:1766851. 10.1080/15592324.2020.1766851 32408840 PMC8570702

[B77] Smaczniak C. Immink R. G. Angenent G. C. Kaufmann K. (2012). Developmental and evolutionary diversity of plant MADS-domain factors: insights from recent studies. *Development* 139 3081–3098. 10.1242/dev.074674 22872082

[B78] Soltis D. E. Chanderbali A. S. Kim S. Buzgo M. Soltis P. S. (2007). The ABC Model and its Applicability to Basal Angiosperms. *Ann. Bot.* 100 155–163. 10.1093/aob/mcm117 17616563 PMC2735328

[B79] Sun G. Xu Y. Liu H. Sun T. Zhang J. Hettenhausen C. (2018). Large-scale gene losses underlie the genome evolution of parasitic plant *Cuscuta australis*. *Nat. Commun*. 9:2683. 10.1038/s41467-018-04721-8 29992948 PMC6041341

[B80] Tapia-López R. Garciía-Ponce B. Dubrovsky J. G. Garay-Arroyo A. Pérez-Ruiíz R. V. Kim S.-H. (2008). An AGAMOUS-Related MADS-Box Gene, XAL1 (AGL12), Regulates Root Meristem Cell Proliferation and Flowering Transition in *Arabidopsis*. *Plant Physiol.* 146 1182–1192. 10.1104/pp.107.108647 18203871 PMC2259045

[B81] Theissen G. Saedler H. (2001). Plant biology. Floral quartets. *Nature* 409 469–471. 10.1038/35054172 11206529

[B82] Theißen G. Kim J. T. Saedler H. (1996). Classification and phylogeny of the MADS-box multigene family suggest defined roles of MADS-box gene subfamilies in the morphological evolution of eukaryotes. *J. Mol. Evol.* 43 484–516. 10.1007/BF02337521 8875863

[B83] Theißen G. Melzer R. Rümpler F. (2016). MADS-domain transcription factors and the floral quartet model of flower development: linking plant development and evolution. *Development* 143 3259–3271. 10.1242/dev.134080 27624831

[B84] Tian Y. Dong Q. Ji Z. Chi F. Cong P. Zhou Z. (2015). Genome-wide identification and analysis of the MADS-box gene family in apple. *Gene* 555 277–290. 10.1016/j.gene.2014.11.018 25447908

[B85] Wagner A. (2000). Robustness against mutations in genetic networks of yeast. *Nat. Genet.* 24 355–361. 10.1038/74174 10742097

[B86] Wang R. Ming M. Li J. Shi D. Qiao X. Li L. (2017). Genome-wide identification of the MADS-box transcription factor family in pear (*Pyrus bretschneideri*) reveals evolution and functional divergence. *Peer J* 5:e3776. 10.7717/peerj.3776 28924499 PMC5598432

[B87] Wang S. Liang H. Xu Y. Li L. Wang H. Sahu D. N. (2021). Genome-wide analyses across Viridiplantae reveal the origin and diversification of small RNA pathway-related genes. *Commun. Biol.* 4:412. 10.1038/s42003-021-01933-5 33767367 PMC7994812

[B88] Wang Y. Liu K. Liao H. Zhuang C. Ma H. Yan X. (2008). The plant WNK gene family and regulation of flowering time in *Arabidopsis*. *Plant Biol.* 10 548–562. 10.1111/j.1438-8677.2008.00072.x 18761494

[B89] Wang Y. Zhang J. Hu Z. Guo X. Tian S. Chen G. (2019). Genome-Wide Analysis of the MADS-Box Transcription Factor Family in *Solanum lycopersicum*. *Int. J. Mol. Sci.* 20:2961. 10.3390/ijms20122961 31216621 PMC6627509

[B90] Weigel D. Meyerowitz E. M. (1994). The ABCs of floral homeotic genes. *Cell* 78 203–209. 10.1016/0092-8674(94)90291-7 7913881

[B91] Westwood J. H. Yoder J. I. Timko M. P. Depamphilis C. W. (2010). The evolution of parasitism in plants. *Trends Plant Sci.* 15 227–235. 10.1016/j.tplants.2010.01.004 20153240

[B92] Xu C. Q. Liu H. Zhou S. S. Zhang D. X. Zhao W. Wang S. (2019). Genome sequence of *Malania oleifera*, a tree with great value for nervonic acid production. *Gigascience* 8:giy164. 10.1093/gigascience/giy164 30689848 PMC6377399

[B93] Xu W. Fiume E. Coen O. Pechoux C. Lepiniec L. Magnani E. (2016). Endosperm and Nucellus Develop Antagonistically in *Arabidopsis* Seeds. *Plant Cell* 28 1343–1360. 10.1105/tpc.16.00041 27233529 PMC4944409

[B94] Yang T. Sahu S. K. Yang L. Liu Y. Mu W. Liu X. (2022). Comparative Analyses of 3,654 Plastid Genomes Unravel Insights Into Evolutionary Dynamics and Phylogenetic Discordance of Green Plants. *Front. Plant Sci*. 13:808156. 10.3389/fpls.2022.808156 35498716 PMC9038950

[B95] Yoo S. K. Lee J. S. Ahn J. H. (2006). Overexpression of AGAMOUS-LIKE 28 (AGL28) promotes flowering by upregulating expression of floral promoters within the autonomous pathway. *Biochem. Biophys. Res. Commun.* 348 929–936. 10.1016/j.bbrc.2006.07.121 16899218

[B96] Yoshida S. Kim S. Wafula E. K. Tanskanen J. Kim Y.-M. Honaas L. (2019). Genome sequence of *Striga asiatica* provides insight into the evolution of plant parasitism. *Curr. Biol*. 29 3041–3052.e4. 10.1016/j.cub.2019.07.086 31522940

[B97] Yun J. Kim Y. S. Jung J. H. Seo P. J. Park C. M. (2012). The AT-hook motif-containing protein AHL22 regulates flowering initiation by modifying FLOWERING LOCUS T chromatin in *Arabidopsis*. *J. Biol. Chem.* 287 15307–15316. 10.1074/jbc.M111.318477 22442143 PMC3346147

