# Supplementary material for: Genome-Wide Analysis of the MADS-Box Gene Family in Holoparasitic Plants (Balanophora subcupularis and Balanophora fungosa var. globosa)
Source: Front Plant Sci. 2022 May 31;13:846697. doi: 10.3389/fpls.2022.846697 (PMC9197559; doi:10.3389/fpls.2022.846697)
Supplement: Supplementary file 1 [file Data_Sheet_1.docx]

Supplementary Material

# Supplementary Figures and Tables

## Supplementary Figures

##
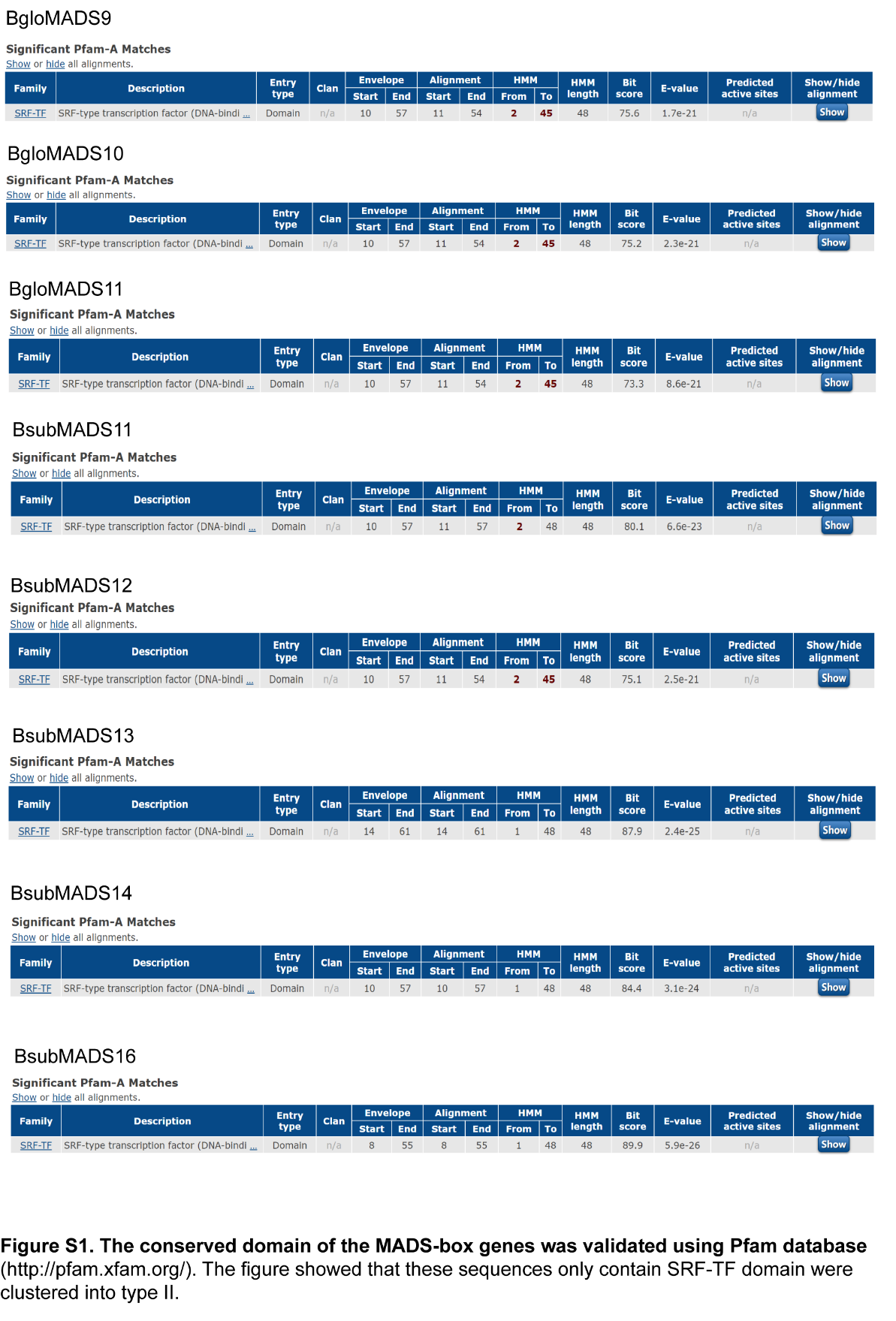


**Supplementary Figure S1.** The conserved domain of the MADS-box genes was validated using Pfam database (http://pfam.xfam.org/). The figure showed that these sequences only contain SRF-TF domain were clustered into type II.


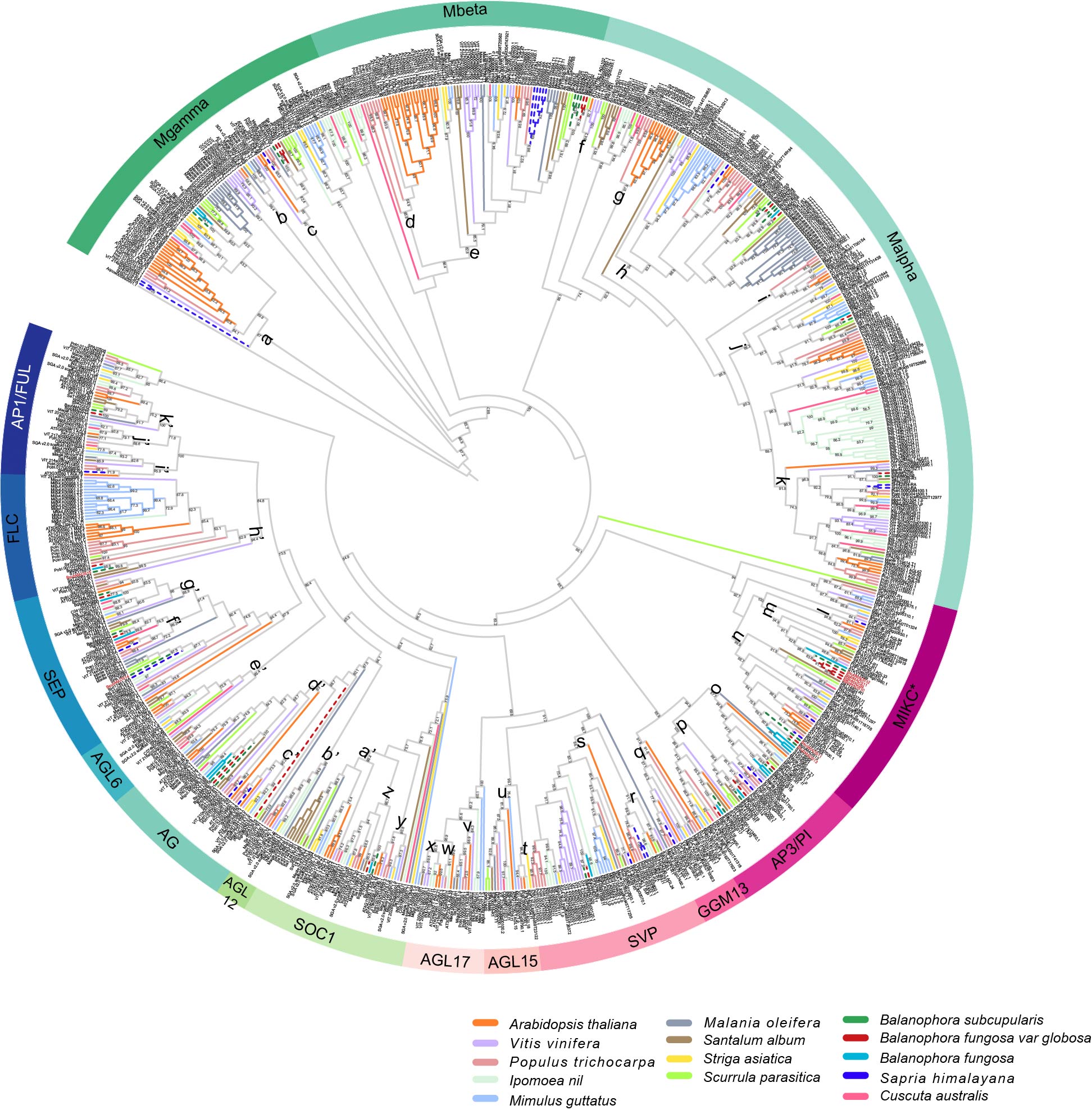


**Supplementary Figure 2.** Phylogenetic tree of MADS-box genes in *B. subcupularis, B. fungosa* var*. globosa* and reference species. Different species are represented in different colors. Orange, green, blue, and red dotted lines represent *Arabidopsis, B. subcupularis, S. himalayana B. fungosa* var. *globosa*, respectively. Using *Arabidopsis* as a reference, orthogroups are divided in more detail to judge gene loss based on homology (see methods for more details). Orthogroups are denoted by a-z, a’-k’. Gray areas indicate branches that cannot be classified. Labels on the red are non-K domain genes that were clustered into type II, and the Bootstraps are ≥50.


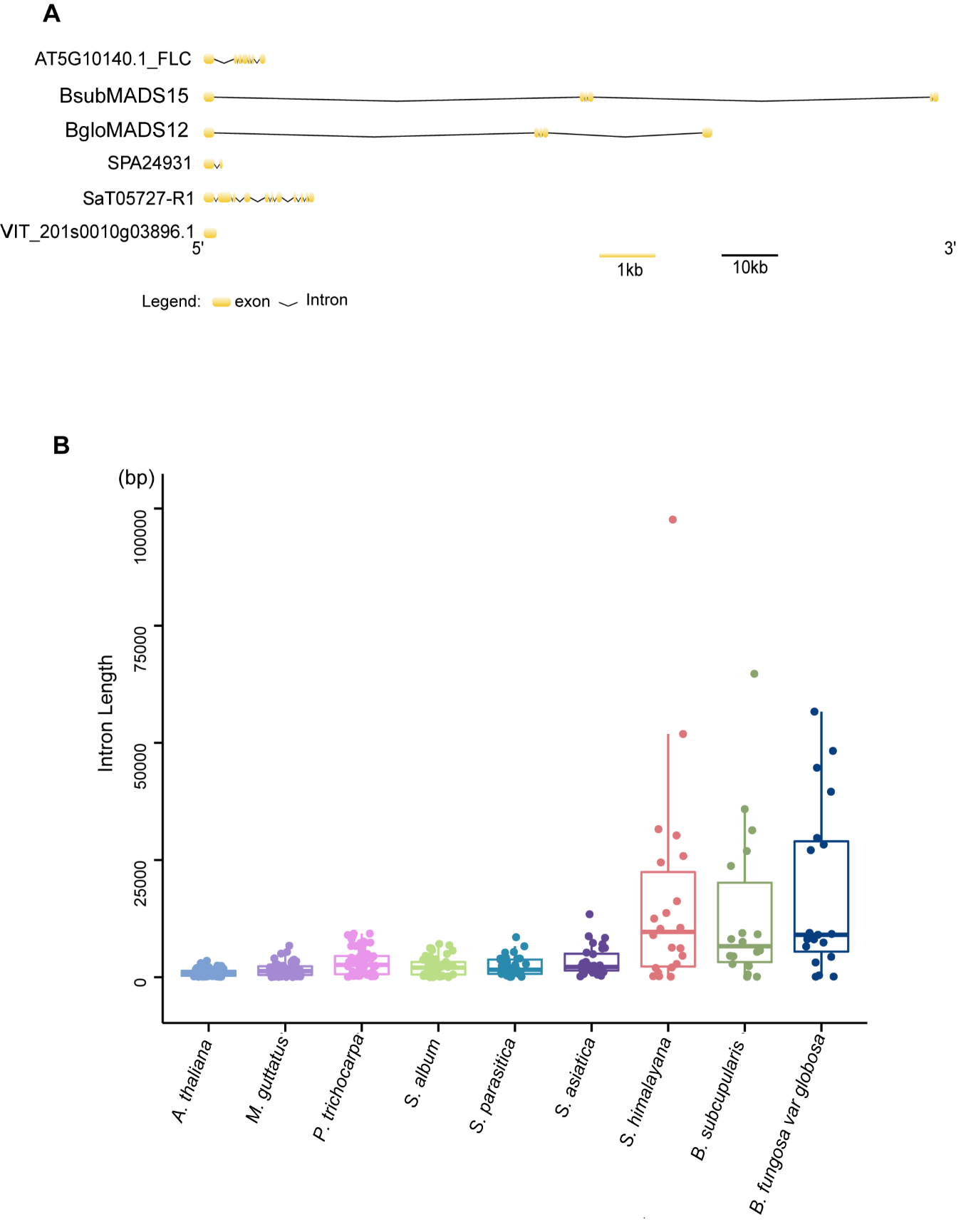


**Supplementary Figure 3.** Intron length of *B. subcupularis, B. fungosa* var*. globosa* and reference species. **(A)** The gene structure of *FLC* gene in *B. subcupularis, B. fungosa* var*. globosa* and reference species (*S. parasitica, S. album, A. thaliana, V. vinifera*). The yellow box shows exon, and the curve shows intron. **(B)** Maximum intron length of per MADS-box gene in each species. The X-axis indicates species, the Y-axis indicates the length of intron.

**
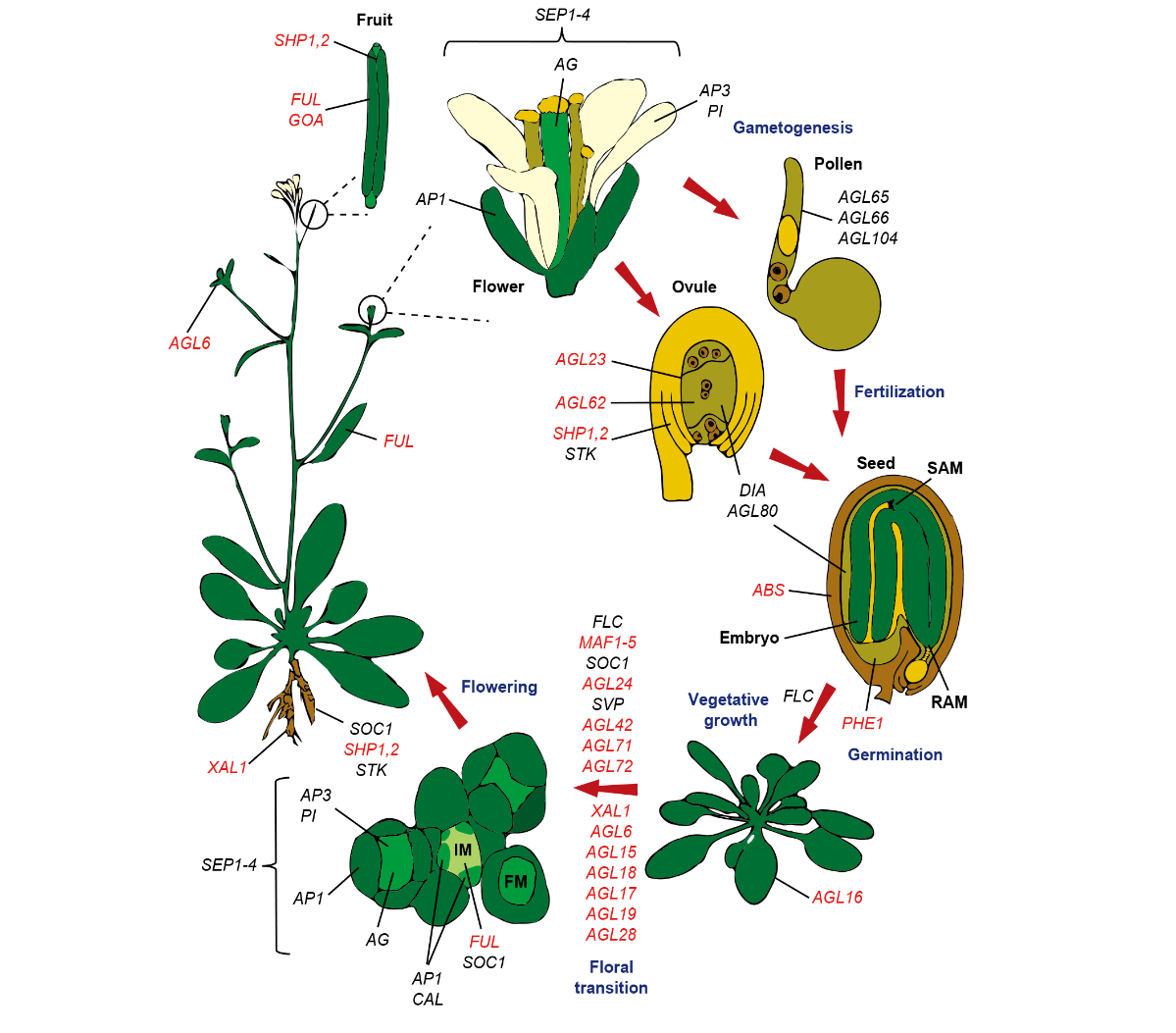
**

**Supplementary Figure 4.** The functions of MADS-box genes in growth and development of *Arabidopsis*. This figure was modified from previous study (Smaczniak et al., 2012). Genes marked in red indicated lost and black indicated retained in *Balanophora*.

**
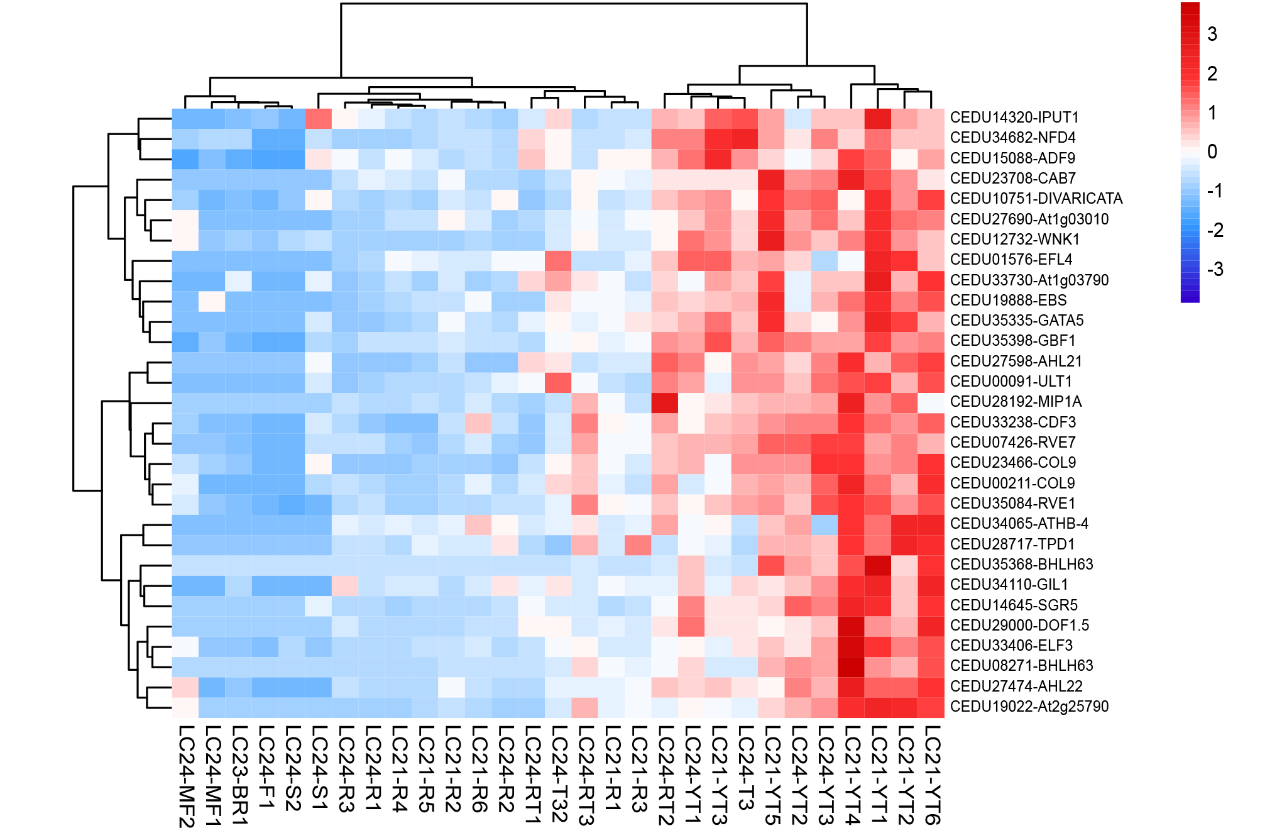
**

**Supplementary Figure 5.** Expression analysis of flowering related genes in the host of *B. fungosa* var*. globosa*. The red and blue colors indicate the expression levels of host genes from high to low, and white indicates the median expression level in the heatmap. Transcript information: (T1 stage) young tuber in stage 1 with diameter <8mm (LC21-YT1, LC21-YT2, LC21-YT3), (T2 stage) young tuber in stage 2 with diameter <15mm (LC21-YT4, LC21-YT5, LC21-YT6), proximal host root (LC21-R1, LC21-R2, LC21-R3), distal host root (LC21-R4, LC21-R5, LC21-R6), (T3 stage) young tuber in stage 3 without visual inflorescence (LC24-YT1, LC24-YT2, LC24-YT3), (T4 stage) seedling in stage 4 with inflorescence were divided into tuber (LC24-T3, LC24-T32), male and female inflorescence (LC24-MF1, LC24-MF2, LC24-F1), bracts (LC23-BR1), inflorescence stem (LC24-S1, LC24-S2), distal host root of grown seedling (LC24-R1, LC24-R2, LC24-R3).

# Supplementary Tables

**Supplementary Table 1 |** Data on all species used in our analysis

**Supplementary Table 2 |** List of 48 MADS-box genes identified in *B. subcupularis* and *B. fungosa* var*. globosa* (aa, amino acids)

**Supplementary Table 3 |** Classification of 14 species based on phylogenetic relationships

**Supplementary Table 4 |** MADS-box clades preserved in *Balanophora* and *S*. *himalayana* in the MRCA of extant angiosperms

**Supplementary Table 5 |** Number of MADS-box gene introns in 12 species based on annotation information

**Supplementary Table 6 |** Length of the first exon of MADS-box in *B. subcupularis* and *B. fungosa* var*. globosa*

**Supplementary Table 7 |** Conserved motifs identified in *B. subcupularis* and *B. fungosa* var*. globosa* MADS-box proteins using the MEME motif search tool (aa, amino acids)

**Supplementary Table 8 |** Gene number of flower meristem and flower-related genes in *Balanophora* and reference species
